# Bayesian and non-bayesian approaches for estimating the Epanechnikov-Weibull distribution with applications in engineering and electronics data

**DOI:** 10.1038/s41598-026-52478-8

**Published:** 2026-06-08

**Authors:** T. S. Taher, H. M. Barakat, H. N. Alqifari, M. A. Alawady, G. M. Mansour

**Affiliations:** 1 Department of Mathematics, Faculty of Science, Zagazig University, Zagazig, 44519 Egypt; 2 Department of Statistics and Operations Research, College of Science, Qassim University, Buraydah, 51482 Saudi Arabia

**Keywords:** Epanechnikov-Weibull distribution, Maximum likelihood estimation, Bayesian estimation, Simulation, Extropy, Weighted extropy, Engineering, Mathematics and computing

## Abstract

Statistical models are widely acknowledged as effective in representing practical scenarios. Nevertheless, it is imperative to recognize that they can sometimes prevent optimal fitting in certain cases. As a result of this awareness, researchers have investigated improved and more efficient probability distributions. This study examines the generalized order statistics (GOSs) of the Epanechnikov-Weibull distribution (EpWD). Statistical features, such as the moment-generating function, the *r*th L-moment, and trimmed L-moments for this model, are derived. Furthermore, we construct estimation techniques for the model parameters employing maximum likelihood estimation (MLE) and Bayesian approaches. Asymptotic confidence intervals (CIs) are formulated by obtaining explicit formulations for the Fisher information matrix (FIM). A Monte Carlo simulation is conducted to assess the efficacy of the suggested estimators using progressively type-II censored samples. This distribution is also analyzed for its extropy and weighted extropy as information-theoretic measures. The practical applicability of the theoretical findings is illustrated through numerical examples that utilize electronic and engineering datasets.

## Introduction

Kamps^1^ proposed GOSs as a unified model for ascendingly ordered random variables (RVs). The GOS model is commonly used in reliability analysis, survival research, and actuarial science to assess life expectancy, failure times, and extreme events. One significant feature of GOSs is their ability to handle censored or truncated data, making them highly valuable in medical statistics and quality control. Furthermore, GOSs are crucial for estimating the parameters of probability distributions, such as the exponential, Weibull, and Pareto distributions. Modeling complicated data patterns in economics, engineering, and environmental research becomes easier with GOSs thanks to their incorporation of weight functions and dependent structures. Moreover, their versatility enables robust inference in non-parametric and semi-parametric settings, improving our understanding of distributional properties. Characterizations of distributions based on GOSs were studied by several authors, see Abd Elgawad et al.^2,3^, Ahsanullah^4^, Beg and Ahsanullah^5^, Cramer et al.^6^, Nagy et al.^7^, Shah et al.^8^, Shah and Barakat^9^, and Tavangar and Asadi^10^.

Kamps^1^ defined a crucial submodel of GOSs known as $m-$GOSs, which contains the most significant models of ordered RVs, including order statistics (OSs), lower and upper record values, $k-$records, sequential OSs, and progressive type II censoring with a constant scheme. Let $F_Y$ be the distribution function (DF) of a continuous RV *Y*. Furthermore, let $f_Y$ denote its probability density function (PDF). Fix a shape parameter $m \ge -1$ and $\kappa> 0.$ The ordered RVs $Y_{(1,n,m, \kappa )} \le Y_{(2,n,m, \kappa )} \le \cdots \le Y_{(n,n,m, \kappa )},~n \in \mathbb {N},$ are called the *m*-GOS if their joint PDF (JPDF) is given by

1 $$\begin{aligned} f_{1,2,\dots ,n:n}^{(m,\kappa )}(y_1,\dots ,y_n) = \left( \prod _{j=1}^{n} \gamma _j \right) \left( \prod _{j=1}^{n-1} \overline{F}_Y^m(y_j) f_Y(y_j) \right) \overline{F}_Y^{\,\kappa - 1}(y_n) f_Y(y_n),~ y_1 \le \cdots \le y_n, \end{aligned}$$

where $F^{-1}_{}(1)\ge y_n\ge ...\ge y_1\ge F^{-1}_{}(0),~\overline{F}_Y(y) = 1 - F_Y(y)$ is the survival function (SF), and $\gamma _j=\kappa +(n-j)(m+1)>0, \;j=1,2,...,n$ (note that $\gamma _n=\kappa$). The marginal PDF of *r*th $m-$GOS, $1\le r\le n,$ is given by using (1) (cf. Kamps^1^,)

2 $$\begin{aligned} f_{Y(r,n,m,\kappa )}(y)=\frac{C_{r-1}}{(r-1)!}(\overline{F}_Y(y))^{\gamma _{r}-1}f_Y(y)g^{r-1}_{m}(F_Y(y)). \end{aligned}$$

Moreover, the JPDF of *r*th and *s*th $m-$GOSs, $1\le r<s\le n,$ is given by

$$\begin{aligned} f_{Y(r,n,m,\kappa ),Y(s,n,m,\kappa )}(y_1,y_2)= & \frac{C_{s-1}}{(r-1)!(s-r-1)!}\overline{F}_Y^{m}(y_1)f_Y(y_1)g^{r-1}_{m}(F_Y(y_1))\\ \times & [h_{m}(F_Y(y_2))-h_{m}(F_Y(y_1))]^{s-r-1}\overline{F}_Y^{\gamma _{s}-1}(y_2)f_Y(y_2),~y_2> y_1, \end{aligned}$$

where $C_{r-1}=\prod \limits _{i=1}^{r}\gamma _i, \,r=1,2,...,n,\, g_{m}(y)=h_{m}(y)-h_{m}(0),\, y\in [0,1)$ and $h_{m}(y)=\frac{-1}{(m+1)}(1-y)^{m+1},$ if $m\ne -1,$ while $h_{-1}(y)=-\ln (1-y).$

The EpWD was introduced by Alzoubi et al.^11^ by combining the flexibility of the Weibull distribution, which is widely used in reliability engineering, with the Epanechnikov kernel (EPK), known for its efficiency in nonparametric smoothing. This construction yields a flexible model capable of capturing a wide range of skewed behaviors commonly observed in engineering, survival analysis, and environmental sciences; see, for example, Elghaly et al.^12^, Li et al.^13^, and Mansour et al.^14^. Simulation studies and real-data applications presented in the literature demonstrate that the EpWD often outperforms several competing models, including the Weibull and gamma distributions. The DF, PDF, SF, and hazard rate function of this model are, respectively, given by

3 $$\begin{aligned} F_{Y}(y;\alpha ,\lambda )=1+\frac{1}{2}e^{-3\left( \frac{y}{\lambda }\right) ^{\alpha }}-\frac{3}{2}e^{-2\left( \frac{y}{\lambda }\right) ^{\alpha }},~~~\alpha ,\lambda>0,~~~ y>0, \end{aligned}$$

4 $$\begin{aligned} \qquad \qquad f_{Y}(y;\alpha ,\lambda )=\left( \frac{3\alpha }{2\lambda ^{\alpha }}\right) y^{\alpha -1}e^{-2\left( \frac{y}{\lambda }\right) ^{\alpha }}\left( 2-e^{-\left( \frac{y}{\lambda }\right) ^{\alpha }}\right) ,~~~\alpha ,\lambda>0,~~~ y>0, \end{aligned}$$

$$\begin{aligned} R_{Y}(y;\alpha ,\lambda )=\frac{3}{2}e^{-2\left( \frac{y}{\lambda }\right) ^{\alpha }}-\frac{1}{2}e^{-3\left( \frac{y}{\lambda }\right) ^{\alpha }},~~~\alpha ,\lambda>0,~~~ y>0, \end{aligned}$$

and

$$\begin{aligned} \qquad \qquad h_{Y}(y;\alpha ,\lambda )=\frac{\left( \frac{3\alpha }{2\lambda ^{\alpha }}\right) y^{\alpha -1}e^{-2\left( \frac{y}{\lambda }\right) ^{\alpha }}\left( 2-e^{-\left( \frac{y}{\lambda }\right) ^{\alpha }}\right) }{\frac{3}{2}e^{-2\left( \frac{y}{\lambda }\right) ^{\alpha }}-\frac{1}{2}e^{-3\left( \frac{y}{\lambda }\right) ^{\alpha }}},~~~\alpha ,\lambda>0,~~~ y>0, \end{aligned}$$

where $\alpha$ and $~\lambda$ are the shape and scale parameters, respectively.

### Remark 1.1

The EpWD is constructed as a linear combination of two Weibull-type densities derived from the EPK. It is not related to the Pearson Type V family and does not arise as an extension or special case of it. Rather, the EpWD is obtained by embedding the EPK within a Weibull framework, leading to a distinct class of models. This kernel-based structure ensures finite moments of all orders and admits closed-form expressions for key information measures, which are important advantages over many existing flexible distributions.

### Interpretation of the parameters

The EpWD is governed by two positive parameters: the shape parameter $\alpha$ and the scale parameter $\lambda$. Their practical meanings are as follows:

- **Shape parameter** $\alpha$: It determines the behavior of the hazard rate function. When $\alpha = 1$, the hazard rate is nearly constant (exponential-type decay). For $\alpha> 1$, the hazard rate increases with time, reflecting a wear-out phase. For $\alpha < 1$, the hazard rate decreases, which corresponds to infant mortality or early failures.
- **Scale parameter** $\lambda$: It acts as a scaling factor: A larger $\lambda$ stretches the distribution along the time axis, leading to larger failure times, while a smaller $\lambda$ compresses it.
- **Fixed mixture coefficients:** The construction of the EpWD uses the EPK, which imposes fixed coefficients $2$ and $3$ in the linear combination of Weibull-type densities. Consequently, the model does not allow flexible weighting of the two components; this is a limitation of the EpWD compared to more general mixtures.

These interpretations help practitioners relate the estimated parameters directly to the underlying failure mechanisms.

### Clarification of the model’s definition and properties

To ensure clarity and mathematical rigor, we explicitly state the parameter space and discuss key structural properties of the EpWD. The distribution is defined on the support $y>0$ and is characterized by two strictly positive parameters: the shape parameter $\alpha>0$ and the scale parameter $\lambda>0.$

#### Parameter space and identifiability

The parameter space is given by $\Theta =\{(\alpha ,\lambda )\in \mathbb {R}^2:\alpha>0,\ \lambda>0\}.$ The EpWD is identifiable over $\Theta$ in the sense that distinct parameter pairs correspond to distinct distributions. Specifically, if

$$R_Y(y;\alpha _1,\lambda _1)=R_Y(y;\alpha _2,\lambda _2) \quad \text {for all } y>0,$$

then it follows that

$$\left( \frac{y}{\lambda _1}\right) ^{\alpha _1} =\left( \frac{y}{\lambda _2}\right) ^{\alpha _2} \quad \text {for all } y>0,$$

which is only possible when $\alpha _1=\alpha _2$ and $\lambda _1=\lambda _2$. Hence, the model is identifiable; see Serfling^15^.

#### Structural representation

The PDF in (4) may be equivalently expressed as

$$f_Y(y;\alpha ,\lambda ) = \frac{3\alpha }{2\lambda ^{\alpha }} y^{\alpha -1} \left[ 2e^{-2\left( \frac{y}{\lambda }\right) ^{\alpha }} - e^{-3\left( \frac{y}{\lambda }\right) ^{\alpha }} \right] ,$$

which represents a linear combination of two Weibull-type density components with common shape parameter $\alpha$ and scale parameters $2^{-1/\alpha }\lambda$ and $3^{-1/\alpha }\lambda$, respectively. This kernel-induced structure enhances the flexibility of the model in capturing varying degrees of skewness and tail decay.

#### Limiting behavior

The EpWD exhibits the following limiting properties:

- For no finite values of $(\alpha ,\lambda )$ does the EpWD reduce to a standard named distribution such as the exponential, Rayleigh, or Weibull distribution. The density remains a nontrivial linear combination of two Weibull-type kernels for all $\alpha ,\lambda>0$, and this structure does not collapse to a single Weibull component under any admissible parameter choice. This feature distinguishes the EpWD from simpler Weibull extensions and underscores its structural novelty.

#### Regularity conditions for MLE

The EpWD satisfies the standard regularity conditions required for the existence, consistency, and asymptotic normality of the MLEs of the parameter vector $\boldsymbol{\theta }=(\alpha ,\lambda )^{\top }.$ These conditions are verified as follows.

(i) Correct model specification and identifiability. The EpWD is a well-defined parametric family with parameter space $\Theta .$ As established earlier, the model is identifiable over $\Theta$, meaning that distinct parameter values correspond to distinct probability distributions. Identifiability ensures the uniqueness of the true parameter vector.

(ii) Support independent of parameters. The support of the EpWD is $(0,\infty )$ and does not depend on the parameters $\alpha$ and $\lambda$. This satisfies a key regularity requirement and allows differentiation under the integral sign when deriving likelihood-based properties.

(iii) Smoothness of the likelihood function. The PDF $f_Y(y;\alpha ,\lambda )$ is continuously differentiable with respect to $\alpha$ and $\lambda$ for all $y>0$ and $(\alpha ,\lambda )\in \Theta$. Moreover, the first- and second-order partial derivatives of the log-likelihood function exist and are finite almost surely.

(iv) Finite Fisher information. The score functions with respect to $\alpha$ and $\lambda$ have finite second moments, implying that the FIM exists and is finite for all $(\alpha ,\lambda )\in \Theta .$ This follows from the Weibull-type exponential decay of the density, which guarantees the finiteness of moments of all orders.

(v) Dominated convergence and interchange of differentiation and integration. Owing to the exponential tail behavior of the EpWD, the likelihood derivatives are dominated by integrable functions. Consequently, differentiation under the integral sign is justified, ensuring the validity of likelihood-based asymptotic arguments.

(vi) Interior true parameter. The true parameter vector $\boldsymbol{\theta }_0=(\alpha _0,\lambda _0)^{\top }$ is assumed to lie in the interior of the parameter space $\Theta$, excluding boundary cases such as $\alpha \rightarrow 0$ or $\lambda \rightarrow 0.$

Taken together, these properties ensure that the regularity conditions commonly required for maximum likelihood inference are satisfied. As a result, the MLEs of $\alpha$ and $\lambda$ are consistent, asymptotically normal, and efficient, with asymptotic covariance matrix given by the inverse of the FIM. For more details, see Lehmann and Casella^16^.

### Motivation and distinction of the EpWD

The EpWD was introduced by Alzoubi et al.^11^ to address a fundamental limitation of the classical Weibull distribution, namely its relatively rigid tail behavior governed by a single shape parameter $\alpha$. In many engineering and survival applications, this limitation restricts the ability of the Weibull model to adequately capture heavy-tailed and highly skewed data patterns (Li et al., 2025).

The EpWD overcomes this limitation through a structurally distinct construction: it embeds the EPK, a second-order polynomial kernel that is optimal in the mean squared error sense for nonparametric density estimation, directly into the Weibull framework. As a result, the EpWD density can be expressed as a linear combination of two Weibull-type components with a common shape parameter $\alpha$ and scale parameters $2^{-1/\alpha }\lambda$ and $3^{-1/\alpha }\lambda .$ This kernel-induced representation fundamentally modifies the tail behavior and yields a class of models that is not obtainable through standard parametric extensions of the Weibull distribution.

It is important to emphasize that the EpWD is not a reparameterization or special case of any classical family (such as exponential, Rayleigh, Weibull, or Pearson-type distributions), but rather represents a genuinely new construction combining parametric and kernel-based ideas.

To further clarify its contribution, we highlight the following distinguishing features of the EpWD in comparison with existing flexible distributions (e.g., exponentiated Weibull, beta Weibull):

- **Kernel-based construction:** Unlike purely parametric generalizations, the EpWD is derived by embedding the EPK into a Weibull structure. This provides a principled bridge between nonparametric kernel methodology and parametric modeling, yielding a tractable yet flexible distribution.
- **Enhanced tail flexibility:** The linear combination structure generates a broader class of tail behaviors than the standard Weibull model. In particular, it allows improved modeling of heavy-tailed and highly skewed data, as confirmed by the simulation study (Section Simulation study) and real data analyses (Section Application of real data).
- **Closed-form information measures:** The EpWD admits explicit expressions for extropy and weighted extropy (Section Extropy and weighted extropy measures). Such closed-form results are typically unavailable for many flexible distributions, where numerical integration is required.
- **Full moment tractability:** The EpWD possesses finite moments of all orders (Remark 2.2), and its moment-generating function (MGF) is available in explicit form (Theorem 2.1), enabling analytical derivation of key statistical quantities.
- **Non-reducibility:** The model does not collapse to any standard distribution for finite parameter values. This distinguishes it from many extensions that simply embed simpler models as special cases.
- **Comparison with alternative kernels:** While other kernels (e.g., uniform or triangular) could in principle be used, they lead to densities that are less smooth or involve discontinuities and typically do not yield tractable closed-form information measures. In contrast, the EPK provides an optimal balance between smoothness, analytical tractability, and statistical efficiency.
- **Empirical support:** Recent studies have demonstrated the effectiveness of Epanechnikov-based constructions across a range of applications. For example, Elghaly et al.^12^ and Mansour et al.^14^ reported improved model fit over classical distributions in reliability and lifetime data. Additional developments (e.g., Epanechnikov–Burr-XII and wrapped Epanechnikov models) further illustrate the flexibility and applicability of this framework.

These structural, theoretical, and empirical properties collectively establish the EpWD as a distinct and practically useful alternative to existing Weibull generalizations and other flexible distributions for modeling complex lifetime data. Taken together, these features establish the EpWD as a novel, well-motivated, and practically useful alternative to standard and recently proposed lifetime distributions.

Let *Y* be a non-negative RV with an absolutely continuous DF $F_Y(\cdot )$ and PDF $f_Y(\cdot ).$ Then, the differential extropy is defined as

5 $$\begin{aligned} J(Y) = \frac{-1}{2}\int _{0}^{\infty }f^{2}_{Y}(y)dy=\frac{-1}{2}\int _{0}^{1}f_Y(F_Y^{-1}(u))du. \end{aligned}$$

Extropy, introduced by Lad et al.^17^ as a complementary dual to entropy, offers an alternative perspective on distributional complexity. While entropy $H(Y) = -\int _{0}^{\infty } f_Y(y)\ln f_Y(y)dy$ measures the average unpredictability or disorder in a distribution, extropy quantifies the distribution’s concentration in terms of its squared density. Low extropy (in absolute value) indicates a highly concentrated distribution (e.g., near a point mass), while high absolute extropy suggests greater dispersion. This duality, established with mathematical rigor by Lad et al.^17^, enriches the information measurement landscape by highlighting complementary aspects of uncertainty. In reliability and survival analysis, where understanding both central tendency and tail behavior is critical, extropy provides a valuable tool for assessing how tightly failure times cluster around typical values versus how they spread in the tails. Despite its theoretical appeal, only a few studies have examined extropy within the framework of GOSs, most notably Abd-Elgawad et al. (2024) and Alawady et al.^18,19^.

The weighted extropy (WEX) measure, established by Abdul Sathar and Nair^20^, generalizes the ordinary extropy stated in (5) by integrating a non-negative weight function, *w*(*y*), into its formulation. The WEX facilitates the accentuation or attenuation of particular areas within a probability distribution. This increased flexibility yields more nuanced insights into the nature of uncertainty and the configuration of the distribution. In the literature, only a limited number of studies examine the concept of WEX in GOSs, particularly Aldallal et al.^21^. When the weight function is $w(y)=y$, the WEX measure accentuates bigger values of the RV, hence emphasizing the upper tail of the distribution. Balakrishnan et al.^22^ offer a comprehensive examination of WEX, including instances like $w(y)=y,$ wherein the authors established the WEX idea and investigated its theoretical attributes and prospective applications. In this specific case, the WEX is defined as

$$\begin{aligned} J^{(\omega )}(Y)=\frac{-1}{2}\int _{0}^{\infty }yf^{2}_Y(y)dy. \end{aligned}$$

Trimmed L-moments (TL-moments), introduced by Elamir and Seheult^23^, provide a robust alternative to conventional L-moments and classical moments. By trimming a specified number of extreme observations from both ends of the sample, TL-moments offer improved resistance to outliers while preserving many of the desirable properties of L-moments, such as linearity and interpretability. The *r*th generalized TL-moment, where $t_1$ and $t_2$ denote the number of smallest and largest values trimmed, respectively, is defined as:

6 $$\begin{aligned} L^{(t_1,t_2)}_{r}=\frac{1}{r}\sum _{i=0}^{r-1}\left( {\begin{array}{c}r-1\\ i\end{array}}\right) (-1)^{i}~E(X_{r+t_1-i:r+t_1+t_2}),~ t_1, t_2,\; r=1,2,..., \end{aligned}$$

where $E\left( X_{r+t_1-i:r+t_1+t_2}\right)$ denotes the expected value of the $(r+t_1-i)$th OS from a random sample of size $r+t_1+t_2$. When $t_1=t_2=0$, the equation (6) reduces to the original L-moments as defined by Hosking^24^.

This paper is organized as follows: In Section Characterization results for the EpWD based on *m*−GOS, we derive several statistical properties of the EpWD based on *m*-GOS, including the MGF, the *r*th L-moment, and TL-moment. Section Extropy and weighted extropy measures investigates the extropy and WEX measures for the EpWD under the *m*-GOS framework. In Section Two different estimation methods, we present both MLE and Bayesian approaches for estimating the model parameters based on progressively type-II censored samples. Additionally, we derive an explicit expression for the FIM, which is used to construct CIs. Section Simulation study provides results from a Monte Carlo simulation study conducted to assess and compare the performance of the proposed estimators. Finally, Section Application of real data presents applications of the proposed methods to two real-world datasets. In conclusion, the paper is finalized in Section Conclusion.

## Characterization results for the EpWD based on $m-$GOS

We start this section by obtaining an explicit expression of the MGF for the marginal moments of EpWD based on $m-$GOSs.

### Theorem 1

*Let* *Y* *follow the EpWD. Then the MGF for this RV under* $m-$*GOS,* $m\ne -1,$ *is given as*

7 $$\begin{gathered} \mathrm{M} _{{(r,n,m,\kappa )}} (t) = \frac{{C_{{r - 1}} }}{{(m + 1)^{{r - 1}} (r - 1)!}}\sum\limits_{{w = 0}}^{{r - 1}} {\sum\limits_{{p = 0}}^{\infty } {\sum\limits_{{u = 0}}^{{\eta _{w} }} {( - 1)^{{w + u}} } } } \left( {\begin{array}{*{20}c} {r - 1} \\ w \\ \end{array} } \right)\left( {\begin{array}{*{20}c} {\eta _{w} } \\ u \\ \end{array} } \right)\left( {\frac{1}{3}} \right)^{u} \left( {\frac{3}{2}} \right)^{{\eta _{w} }} \hfill \\ \quad \quad \frac{{(t\lambda )^{p} (2\eta _{w} + u)^{{\frac{{ - p}}{\alpha }}} \frac{p}{\alpha }\Gamma (\frac{p}{\alpha })}}{{\eta _{w} p!}}, \hfill \\ \end{gathered}$$

*where* $\eta _w=\gamma _r+w(m+1)$ *and* *m* *and* $\kappa$ *are chosen such that the* $\eta _w$ *is an integer*.

**Proof **Starting with (2), (3), and (4), we get

$$\begin{aligned} \textsf {M}_{(r,n,m,\kappa )}(t)\!=\!\int _{0}^{\infty }e^{ty}f_{Y(r,n,m,\kappa )}(y)dy =\frac{C_{r-1}}{(r-1)!}\int _{0}^{\infty }e^{ty}(\overline{F}_Y(y))^{\gamma _{r}-1}f_Y(y)g^{r-1}_{m}(F_Y(y))dy. \end{aligned}$$

Apply the binomial expansion, we obtain

$$g_m^{r-1}(F_Y(y))=\frac{1}{(m+1)^{r-1}}\sum _{w=0}^{r-1}\left( {\begin{array}{c}r-1\\ w\end{array}}\right) (-1)^w (\overline{F}_Y(y))^{w(m+1)}.$$

Substituting into the MGF expression yields

$$\operatorname {M}_{(r,n,m,\kappa )}(t)=\frac{C_{r-1}}{(m+1)^{r-1}(r-1)!}\sum _{w=0}^{r-1}\left( {\begin{array}{c}r-1\\ w\end{array}}\right) (-1)^w \int _0^\infty e^{ty}(\overline{F}_Y(y))^{\gamma _r+w(m+1)-1}f_Y(y)\,dy.$$

By using the power series expression $e^{ty}=\sum _{p=0}^\infty \frac{t^p}{p!}y^p,$ we get

$$\operatorname {M}_{(r,n,m,\kappa )}(t)=\frac{C_{r-1}}{(m+1)^{r-1}(r-1)!}\sum _{w=0}^{r-1}\sum _{p=0}^\infty \left( {\begin{array}{c}r-1\\ w\end{array}}\right) (-1)^w\frac{t^p}{p!} \int _0^\infty y^{p}(\overline{F}_Y(y))^{\gamma _r+w(m+1)-1}f_Y(y)\,dy.$$

Using part-integration, we set

$$\operatorname {M}_{(r,n,m,\kappa )}(t)=\frac{p C_{r-1}}{\eta _w(m+1)^{r-1}(r-1)!}\sum _{w=0}^{r-1}\sum _{p=0}^\infty \left( {\begin{array}{c}r-1\\ w\end{array}}\right) (-1)^w\frac{t^p}{p!} \int _0^\infty y^{p-1}(\overline{F}_Y(y))^{\eta _w}\,dy.$$

Using the explicit forms $\overline{F}_Y(y)=R_{Y}(y)=\frac{3}{2}e^{-2(y/\lambda )^\alpha }-\frac{1}{2}e^{-3(y/\lambda )^\alpha },$ we obtain

$$(\overline{F}_Y(y))^{\eta _w}=\Bigl (\tfrac{3}{2} e^{-2z}-\tfrac{1}{2} e^{-3z}\Bigr )^{\eta _w},$$

where $z=(y/\lambda )^\alpha$. Since $\eta _w$ is an integer, we expand

$$\Bigl (\tfrac{3}{2} e^{-2z}-\tfrac{1}{2} e^{-3z}\Bigr )^{\eta _w}=\sum _{u=0}^{\eta _w}\left( {\begin{array}{c}\eta _w\\ u\end{array}}\right) \Bigl (\tfrac{3}{2} e^{-2z}\Bigr )^{\eta _w-u}\Bigl (-\tfrac{1}{2} e^{-3z}\Bigr )^u.$$

Combining exponential terms gives $e^{-2(\eta _w-u)z}e^{-3uz}=e^{-(2\eta _w+u)z},$ which, after rearrangement, can be written in the form $e^{-(2\eta _w+u)(y/\lambda )^\alpha },$ with the constant terms absorbed into the coefficients. Thus, the integral reduces to

$$\begin{aligned} \int _0^\infty y^{p-1} e^{-(2\eta _w+u)(y/\lambda )^\alpha }\,dy. \end{aligned}$$

Using the substitution $s=(y/\lambda )^\alpha$, this integral evaluates to

$$\frac{\lambda ^{p}}{\alpha }(2\eta _w+u)^\frac{-p}{\alpha } \Gamma \!\left( \frac{p}{\alpha }\right) .$$

Substituting back and rearranging coefficients yields the expression stated in the theorem.

### Remark 2.1

To enhance the accessibility of Theorem 2.1, we provide a detailed verification that $\operatorname {M}_{(r,n,m,\kappa )}(0) = 1,$ which serves as a useful check on the correctness of the derived expression. Setting $t = 0$ in equation (2.1), all terms with $p \ge 1$ vanish because they contain factors of $t^p.$ The triple sum therefore collapses to the single term $p = 0,$ yielding:

$$\begin{aligned} \operatorname {M}_{(r,n,m,\kappa )}(0) = \frac{C_{r-1}}{(m+1)^{r-1}(r-1)!} \sum _{w=0}^{r-1} (-1)^w \left( {\begin{array}{c}r-1\\ w\end{array}}\right) \sum _{u=0}^{\eta _w} \left( {\begin{array}{c}\eta _w\\ u\end{array}}\right) \left( -\frac{1}{3}\right) ^u \left( \frac{3}{2}\right) ^{\eta _w} \frac{1}{\eta _w} \lim _{p\rightarrow 0^+} \frac{p}{\alpha } \Gamma \left( \frac{p}{\alpha }\right) . \end{aligned}$$

Using the well-known limit $\lim _{x\rightarrow 0^+} x\Gamma (x) = 1$, the factor involving the gamma function evaluates to $1/\alpha .$ Simplifying the inner *u*-sum via the binomial theorem:

$$\begin{aligned} \sum _{u=0}^{\eta _w} \left( {\begin{array}{c}\eta _w\\ u\end{array}}\right) \left( -\frac{1}{3}\right) ^u = \left( 1 - \frac{1}{3}\right) ^{\eta _w} = \left( \frac{2}{3}\right) ^{\eta _w}, \end{aligned}$$

and noting that $\left( \frac{3}{2}\right) ^{\eta _w} \times \left( \frac{2}{3}\right) ^{\eta _w} = 1,$ the expression reduces to:

$$\begin{aligned} \operatorname {M}_{(r,n,m,\kappa )}(0) = \frac{C_{r-1}}{(m+1)^{r-1}(r-1)!} \sum _{w=0}^{r-1} \frac{(-1)^w}{\eta _w} \left( {\begin{array}{c}r-1\\ w\end{array}}\right) . \end{aligned}$$

Now, set $x = m+1$ and $z = \gamma _r/x$. A known combinatorial identity (see, e.g., Kamps^1^) states that:

$$\begin{aligned} \sum _{w=0}^{r-1} \frac{(-1)^w}{x(z + w)} \left( {\begin{array}{c}r-1\\ w\end{array}}\right) = \frac{(r-1)!}{x(z)_r}, \end{aligned}$$

where $$(z)_r \!= \!z(z+1)\!\cdots \!(z+r-1)$$ is the Pochhammer symbol (rising factorial). Since $$(z)_r \!=\! x^{-r} \prod _{k=0}^{r-1} (\gamma _r$$ $$+ kx),$$ we obtain:

$$\begin{aligned} \sum _{w=0}^{r-1} \frac{(-1)^w}{\eta _w} \left( {\begin{array}{c}r-1\\ w\end{array}}\right) = \frac{(r-1)! (m+1)^{r-1}}{\prod _{k=0}^{r-1} (\gamma _r + k(m+1))}. \end{aligned}$$

Observing that $\gamma _r + k(m+1) = \gamma _{r-k},$ the denominator becomes $\prod _{j=1}^r \gamma _j = C_{r-1}.$ Substituting back:

$$\begin{aligned} \operatorname {M}_{(r,n,m,\kappa )}(0) = \frac{C_{r-1}}{(m+1)^{r-1}(r-1)!} \times \frac{(r-1)! (m+1)^{r-1}}{C_{r-1}} = 1, \end{aligned}$$

which confirms the desired result. This verification not only validates the MGF expression but also illustrates the systematic application of binomial expansions and combinatorial identities that underlie many of the derivations in this section.

### Remark 2.2

For applied researchers, it is important to understand why the MGF exists for all real *t* under the constraints $\alpha , \lambda , \gamma _r> 0.$ Convergence of the series in (7) is ensured by three key factors:

1. **Dominating term:** The factor $(2\eta _w + u)^{-p/\alpha }$ decays exponentially in *p* because $2\eta _w + u \ge \gamma _r> 0$ (since $\eta _w = \gamma _r + w(m+1)$ and $u \ge 0$).
2. **Gamma function growth:** The gamma function $\Gamma (p/\alpha )$ grows slower than the factorial *p*! in the denominator, ensuring that the terms $\Gamma (p/\alpha )/p!$ decay rapidly.
3. **Factorial decay:** The presence of *p*! in the denominator guarantees absolute convergence of the series for all *t*, as the terms decay factorially.

This existence property has two important practical implications. First, it guarantees that all moments of the EpWD under *m*-GOS are finite, which is essential for moment-based estimation and inference. Second, the finiteness of moments ensures the existence of the FIM (see Section "Asymptotic confidence intervals"), which underpins the asymptotic normality of MLEs and the construction of CIs. Thus, while the derivations may appear mathematically dense, they ultimately support the practical inferential methods applied to real data in Section Application of real data.

Now, define $\mu _{(r,n,m,\kappa )}= E(Y_{(r,n,m,\kappa )})$ and $\mu _{(r,n,m,\kappa )}^{(\ell )} = E(Y_{(r,n,m,\kappa )}^\ell ),~\ell =2,3,...~.$ By differentiating $\textsf {M}_{(r,n,m,\kappa )}(t)$ with respect to *t* and evaluation at $t=0,$ we observe that only the terms with $p=1$ survive (as higher-order terms vanish). Thus, we isolate terms where $p=1$ from the triple summation. This gives

8 $$\begin{aligned} \mu _{(r,n,m,\kappa )}=\left. \frac{d}{dt}\textsf {M}_{(r,n,m,\kappa )}(t)\right| _{t=0}\!\!\!=\sum _{w=0}^{r-1}\sum _{u=0}^{\eta _w}\frac{C_{r-1}\left( {\begin{array}{c}r-1\\ w\end{array}}\right) (-1)^{w+u}\left( {\begin{array}{c}\eta _w\\ u\end{array}}\right) \left( \frac{1}{3}\right) ^{u}\!\!\left( \frac{3}{2}\right) ^{\eta _w}\lambda (2\eta _w+u)^{\frac{-1}{\alpha }} \Gamma (\frac{1}{\alpha })}{\alpha (m+1)^{r-1}(r-1)!\eta _w}. \end{aligned}$$

Similarly, the second moment of EpWD under $m-$GOS can be obtained as

9 $$\begin{aligned} \mu ^{(2)}_{(r,n,m,\kappa )}=\left. \frac{d^{2}}{dt^{2}}\textsf {M}_{(r,n,m,\kappa )}(t)\right| _{t=0}\!\!\!=\sum _{w=0}^{r-1}\sum _{u=0}^{\eta _w}\frac{2C_{r-1}\left( {\begin{array}{c}r-1\\ w\end{array}}\right) (-1)^{w+u}\left( {\begin{array}{c}\eta _w\\ u\end{array}}\right) \left( \frac{1}{3}\right) ^{u}\!\!\left( \frac{3}{2}\right) ^{\eta _w} \lambda ^{2}(2\eta _w+u)^{\frac{-2}{\alpha }}\Gamma (\frac{2}{\alpha })}{\alpha (m+1)^{r-1}(r-1)!\eta _w}. \end{aligned}$$

Numerical values for the mean and variance of OSs for the EpWD are obtained by taking $m=0$ and $\kappa =1$ in (8) and (9) for some values of parameters in Table 1. The results in Table 1 are consistent with the property of OSs (i.e. $\sum _{i=1}^{n}\mu _{i:n}=n\mu _{1:1}$) given by David and Nagaraja^25^.

**Table 1:** Mean and variance of EpWD based on OSs.

Mean					Variance		
*n*	*r*	$\lambda =3$ $\alpha =2$	$\lambda =2 ~~\alpha =3$	$\lambda =5 ~~\alpha =4$	$\lambda =3 ~~\alpha =2$	$\lambda =2 ~~\alpha =3$	$\lambda =5 ~~\alpha =4$
1	1	2.05246	1.50712	3.99464	1.0374	0.272771	1.14672
2	1	1.47887	1.2105	3.3882	0.550446	0.179978	0.843989
2	2	2.62605	1.80374	4.60107	0.866338	0.189597	0.713932
3	1	1.21844	1.06342	3.07399	0.379235	0.140873	0.704296
3	2	1.99972	1.50466	4.01663	0.485941	0.128392	0.530988
3	3	2.93922	1.95328	4.89329	0.762315	0.153115	0.549229
4	1	1.06092	0.969402	2.86751	0.290492	0.118179	0.618424
4	2	1.691	1.34546	3.69342	0.347713	0.10289	0.450309
4	3	2.30844	1.66386	4.33984	0.433557	0.103207	0.402738
4	4	3.14948	2.04975	5.07777	0.695061	0.132523	0.461924
5	1	0.952369	0.901915	2.71624	0.235854	0.102994	0.558469
5	2	1.49514	1.23935	3.4726	0.273356	0.0878295	0.400569
5	3	1.98479	1.50464	4.02466	0.315397	0.0832547	0.342061
5	4	2.5242	1.77	4.54997	0.395944	0.0883402	0.332809
5	5	3.3058	2.11968	5.20972	0.64766	0.119113	0.407147
6	1	0.871665	0.850089	2.59816	0.198714	0.0919668	0.513431
6	2	1.35589	1.16105	3.3066	0.226165	0.0775525	0.365416
6	3	1.77366	1.395952	3.8046	0.25138	0.0715956	0.305542
6	4	2.19592	1.61332	4.24471	0.290265	0.071291	0.28173
6	5	2.68834	1.84835	4.7026	0.367956	0.0784515	0.288463
6	6	3.4293	2.17395	5.31115	0.6121	0.109575	0.369161

Now, we derive the *r*th TL−moment for EpWD based on OSs. The *r*th TL−moment can be obtained from (6) and (8) with $m=0,\, \kappa =1$, $n=r+t_1+t_2,$ and $r=r+t_1-i$ as follows:

10 $$\begin{aligned} E(X_{r+t_1-i:r+t_1+t_2})\!=\!\sum _{w=0}^{r+t_1-i-1}\sum _{u=0}^{\eta _w}\frac{(-1)^{w+u}(r\!+\!t_1\!+\!t_2)!\left( {\begin{array}{c}r+t_1-i-1\\ w\end{array}}\right) \left( {\begin{array}{c}\eta _w\\ u\end{array}}\right) \left( \frac{1}{3}\right) ^{u}\!\!\left( \frac{3}{2}\right) ^{\eta _w}\lambda (2\eta _w\!\!+\!\!u)^{\frac{-1}{\alpha }}\Gamma (\frac{1}{\alpha })}{\alpha (r+t_1-i-1)!(n+r+t_1-i-1+w)}. \end{aligned}$$

Then, the *r*th TL−moment of the EpWD is obtained by substituting the expectation (10) in (6).

11 $$\begin{aligned} L^{(t_1,t_2)}_{r}\,= & \,\sum _{w=0}^{r+t_1-i-1}\sum _{u=0}^{\eta _w}\sum _{i=0}^{r-1}(-1)^{i+w+u}\left( {\begin{array}{c}r-1\\ i\end{array}}\right) \frac{(r+t_1+t_2)!\left( {\begin{array}{c}r+t_1-i-1\\ w\end{array}}\right) \left( {\begin{array}{c}\eta _w\\ u\end{array}}\right) \left( \frac{1}{3}\right) ^{u}\,\,\left( \frac{3}{2}\right) ^{\eta _w}}{r\alpha (r+t_1-i-1)!(n+r+t_1-i-1+w)} \nonumber \\ \times & \lambda (2\eta _w+u)^{\frac{-1}{\alpha }}\Gamma (\frac{1}{\alpha }). \end{aligned}$$

Furthermore, the *r*th L−moment can be obtained from (11) with $t_1=t_2=0$ as follows:

12 $$\begin{aligned} L_{r}\,= & \,\sum _{w=0}^{r-i-1}\sum _{u=0}^{\eta _w}\sum _{i=0}^{r-1}(-1)^{i+w+u}\left( {\begin{array}{c}r-1\\ i\end{array}}\right) ~\frac{(r-1)!\left( {\begin{array}{c}r-i-1\\ w\end{array}}\right) \left( {\begin{array}{c}\eta _w\\ u\end{array}}\right) \left( \frac{1}{3}\right) ^{u}\,\left(\frac{3}{2}\right) ^{\eta _w}\lambda (2\eta _w+u)^{\frac{-1}{\alpha }}\Gamma (\frac{1}{\alpha })}{\alpha (r-i-1)!(n+r-i-1+w)}. \end{aligned}$$

The first four L−moment can be obtained from (12) by taking $r=1,2,3,$ and 4, respectively.

## Extropy and weighted extropy measures

In this brief section, we derive the extropy and WEX measures based on the *r*th $m-$GOS from the EpWD.

The extropy for the EpWD, where $\alpha \ge 0.5,$ is given by

$$\begin{aligned} J(Y)= & \frac{-1}{2}\int _{0}^{\infty }f^{2}_{Y}(y)dy =\frac{-1}{2}\int _{0}^{\infty }\left( \left( \frac{3\alpha }{2\lambda ^{\alpha }}\right) y^{\alpha -1}e^{-2\left( \frac{y}{\lambda }\right) ^{\alpha }}\left( 2-e^{-\left( \frac{y}{\lambda }\right) ^{\alpha }}\right) \right) ^{2}dy\nonumber \\= & \frac{-\alpha }{800\lambda }\left( 225\times 4^{\frac{1}{\alpha }}-144\times 5^{\frac{1}{\alpha }}+25\times 6^{\frac{1}{\alpha }}\right) \Gamma \left( 2-\frac{1}{\alpha }\right) . \end{aligned}$$

The extropy for the EpWD based on $m-$GOSs is given by the following theorem:

### Theorem 2

*Let* $\alpha \ge 0.5.$ *Furthermore, let* $Y_{(r,n,m, \kappa )}$ *be the* *rth* $m-$*GOSs for EpWD, then the extropy of* $Y_{(r,n,m, \kappa )}$ *is given by*

$$\begin{aligned} J(Y_{(r,n,m, \kappa )})= & \frac{-1}{2}\int _{0}^{\infty }f^{2}_{Y(r,n,m,\kappa )}(y)dy\nonumber \\= & \sum _{w=0}^{2(r-1)}\sum _{u=0}^{A_w}(-1)^{w+u+1}\frac{C_{r-1}^{2}\left( {\begin{array}{c}2(r-1)\\ w\end{array}}\right) \left( {\begin{array}{c}A_w\\ u\end{array}}\right) (\frac{1}{3})^{u}(\frac{3}{2})^{2+A_w}}{2\left( (m+1)^{r-1}(r-1)!\right) ^{2}} ~\frac{\alpha }{\lambda }\Gamma (2-\frac{1}{\alpha })\nonumber \\ \times & \left[ 4(4+2A_w+u)^{\frac{1}{\alpha }-2}-4(5+2A_w+u)^{\frac{1}{\alpha }-2}+(6+2A_w+u)^{\frac{1}{\alpha }-2}\right] , \end{aligned}$$

*where* $A_w=2(\gamma _r-1)+w(m+1)$ *and* *m* *and* $\kappa$ *are chosen such that the* $A_w$ *is an integer*.

**Proof **The extropy of $Y_{(r,n,m,\kappa )}$ is $J(Y_{(r,n,m,\kappa )})=-\frac{1}{2}\int _0^\infty f_{Y(r,n,m,\kappa )}^2(y)\,dy.$ Using (1.2), we obtain

$$f_{Y(r,n,m,\kappa )}^2(y)=\frac{C_{r-1}^2}{((r-1)!)^2} (\overline{F}_Y(y))^{2(\gamma _r-1)}f_Y^2(y)\,g_m^{2(r-1)}(F_Y(y)).$$

Expanding $~g_m^{2(r-1)}(F_Y(y))$ using the binomial theorem yields

$$g_m^{2(r-1)}(F_Y(y))= \frac{1}{(m+1)^{2(r-1)}} \sum _{w=0}^{2(r-1)}\left( {\begin{array}{c}2(r-1)\\ w\end{array}}\right) (-1)^w (\overline{F}_Y(y))^{w(m+1)}.$$

Thus,

$$f_{Y(r,n,m,\kappa )}^2(y)= \frac{C_{r-1}^2}{(m+1)^{2(r-1)}((r-1)!)^2} \sum _{w=0}^{2(r-1)}\left( {\begin{array}{c}2(r-1)\\ w\end{array}}\right) (-1)^w (\overline{F}_Y(y))^{2(\gamma _r-1)+w(m+1)} f_Y^2(y).$$

Using $\overline{F}_Y(y)=\frac{3}{2}e^{-2z}-\frac{1}{2}e^{-3z}$ and $f_Y(y)=\frac{3\alpha }{2\lambda ^\alpha }y^{\alpha -1}(2e^{-2z}-e^{-3z}),$ with $z=(y/\lambda )^\alpha ,$ we obtain

$$f_Y^2(y)=\left( \frac{3\alpha }{2\lambda ^\alpha }\right) ^2 y^{2(\alpha -1)}(2e^{-2z}-e^{-3z})^2.$$

Expanding $(2e^{-2z}-e^{-3z})^2 = 4e^{-4z}-4e^{-5z}+e^{-6z}$ and similarly expanding $(\overline{F}_Y(y))^{A_w}$ via the binomial theorem (since $A_w$ is an integer), the integrand becomes a finite sum of terms of the form $y^{2(\alpha -1)} e^{-c (y/\lambda )^\alpha },$ for suitable constants $c>0.$ Thus, each term reduces to an integral of the form

$$\begin{aligned} \int _0^\infty y^{2\alpha -2} e^{-c (y/\lambda )^\alpha }\,dy. \end{aligned}$$

Using the substitution $s=(y/\lambda )^\alpha ,$ we obtain

$$\int _0^\infty y^{2\alpha -2} e^{-c (y/\lambda )^\alpha }\,dy=\frac{\lambda ^{2\alpha -1}}{\alpha } c^{-(2-1/\alpha )}\Gamma \!\left( 2-\frac{1}{\alpha }\right) .$$

Substituting these expressions back and summing over all indices yields the desired result.

The WEX for the EpWD is given by

$$\begin{aligned} J^{(\omega )}(Y) = \frac{-1}{2}\int _{0}^{\infty }yf^{2}_{Y}(y)dy=\frac{-1}{2}\int _{0}^{\infty }y\left( \left( \frac{3\alpha }{2\lambda ^{\alpha }}\right) y^{\alpha -1}e^{-2\left( \frac{y}{\lambda }\right) ^{\alpha }}\left( 2-e^{-\left( \frac{y}{\lambda }\right) ^{\alpha }}\right) \right) ^{2}dy =\frac{-53\alpha }{400}. \end{aligned}$$

The WEX for the EpWD based on $m-$GOSs is given by the following theorem:

### Theorem 3

*Let* $Y_{(r,n,m, \kappa )}$ *be the* *rth* $m-$*GOSs for EpWD, then the WEX of* $Y_{(r,n,m, \kappa )}$ *is given by*

$$\begin{aligned} J^{(\omega )}(Y_{(r,n,m, \kappa )})= & \frac{-1}{2}\int _{0}^{\infty }yf^{2}_{Y(r,n,m, \kappa )}(y)dy\nonumber \\= & \sum _{w=0}^{2(r-1)}\sum _{u=0}^{A_w}(-1)^{w+u+1}\frac{\alpha C_{r-1}^{2}\left( {\begin{array}{c}2(r-1)\\ w\end{array}}\right) \left( {\begin{array}{c}A_w\\ u\end{array}}\right) (\frac{1}{3})^{u} \left( \frac{3}{2}\right) ^{2+A_w}}{2\left( (m+1)^{r-1}(r-1)!\right) ^{2}}\nonumber \\ \times & \left[ \frac{4}{(4+2A_w+u)^{2}}-\frac{4}{(5+2A_w+u)^{2}}+\frac{1}{(6+2A_w+u)^{2}}\right] . \end{aligned}$$

**Proof **The WEX of $Y_{(r,n,m,\kappa )}$ is defined as $J^{(\omega )}(Y_{(r,n,m,\kappa )})=-\frac{1}{2}\int _0^\infty y\, f_{Y(r,n,m,\kappa )}^2(y)\,dy.$ Proceeding as in the proof of Theorem 3.1, we expand $f_{Y(r,n,m,\kappa )}^2(y)$ and reduce the integral to a finite sum of terms of the form

$$\begin{aligned} \int _0^\infty y^{2\alpha -1} e^{-c (y/\lambda )^\alpha }\,dy, \end{aligned}$$

for suitable constants $c>0.$ Using the substitution $s=(y/\lambda )^\alpha ,$ we obtain

$$\begin{aligned} \int _0^\infty y^{2\alpha -1} e^{-c (y/\lambda )^\alpha } dy=\frac{\lambda ^{2\alpha }}{\alpha } c^{-2} \Gamma (2). \end{aligned}$$

Since $\Gamma (2)=1$, this simplifies to $\frac{\lambda ^{2\alpha }}{\alpha } c^{-2}.$ Compared to Theorem 3.1, where the corresponding integral involves $y^{2\alpha -2}$ and yields a factor proportional to $c^{-(2-1/\alpha )}$, the additional factor *y* increases the exponent by one, resulting in a $c^{-2}$ dependence. Substituting these expressions back into the expanded form and summing over all indices yields the desired result. The remaining algebraic steps follow directly from those in Theorem 3.1 and are therefore omitted for brevity.

### Interpretation and practical relevance

The extropy and WEX measures derived above offer distinct insights into the structure of the EpWD. To illustrate their interpretation, consider the following:

Scale and units: Both *J*(*Y*) and $J^{(\omega )}(Y)$ are negative-valued for continuous distributions (since $-\frac{1}{2}\int f_Y^2(y)dy < 0$). Their absolute values increase with the dispersion of the distribution. For the EpWD, larger values of the shape parameter $\alpha$ (indicating a steeper failure rate) generally lead to smaller absolute extropy, reflecting increased concentration around the scale parameter $\lambda$.

Tail sensitivity: The WEX $J^{(\omega )}(Y)$ is more sensitive than *J*(*Y*) to changes in the upper tail of the distribution. This is evident from the weight function $w(y)=y,$ which amplifies contributions from larger *y* values. In reliability terms, *J*(*Y*) summarizes overall failure time uncertainty, while $J^{(\omega )}(Y)$ emphasizes uncertainty about late failures-a critical distinction when designing maintenance schedules or warranty periods.

Comparison with entropy: For the EpWD, the differential entropy *H*(*Y*) can be derived and compared with *J*(*Y*). While *H*(*Y*) measures average unpredictability in terms of log-density, *J*(*Y*) measures concentration in terms of squared density. These two measures are complementary: a distribution can have high entropy (high unpredictability) but low absolute extropy (high concentration) if it is concentrated in regions of low density, and vice versa. The simulation results in Section Simulation study (Table 3) demonstrate that $J^{(\omega )}(Y)$ exhibits better estimability than *J*(*Y*), with narrower confidence/credible intervals across all scenarios. This improved precision makes WEX a more reliable tool for inference in practical applications where tail behavior is of interest. 3

Practical implications: The choice between entropy, extropy, and WEX depends on the application context:

- Use entropy when the goal is to quantify overall uncertainty or disorder in a system.
- Use extropy when the focus is on distributional concentration, such as assessing how tightly failure times cluster around a typical value.
- Use WEX when tail events are of primary concern, as in extreme value analysis, reliability of aging systems, or environmental risk assessment.

In the real data applications of Section Application of real data, the estimated extropy and WEX values (Table 8) provide complementary summaries of the fitted EpWD: *J* captures overall concentration, while $J^{(\omega )}$ emphasizes the uncertainty in the upper tail of failure times, offering insights that would not be available from entropy alone.

Connection to risk measures: While not the primary focus of this paper, the EpWD’s finite moments of all orders (Remark 2.2) guarantee the existence of standard risk measures such as Value-at-Risk (VaR) and Tail Value-at-Risk (TVaR). For a given probability level $p \in (0,1),$ VaR$_p$ is simply the *p*-th quantile of the distribution, obtained by inverting Equation (3). In reliability engineering, the $B_{10}$ life-the time by which 10% of a population of units is expected to have failed-corresponds to VaR$_{0.10},$ providing a benchmark for warranty periods and maintenance scheduling. TVaR$_p,$ which measures the expected loss exceeding VaR$_p,$ can be computed as $\text {TVaR}_p = \frac{1}{1-p}\int _{p}^{1} \text {VaR}_u \, du,$ offering a more conservative risk assessment by averaging failures beyond the threshold, which is particularly valuable for safety-critical applications where extreme events are of concern. The WEX $J^{(\omega )}(Y)$ derived in Theorem 3.2 complements these measures by quantifying uncertainty specifically in the upper tail, providing an information-theoretic perspective on extreme failure times. Together, VaR (threshold), TVaR (expected severity), and WEX (tail uncertainty) form a comprehensive toolkit for risk assessment in engineering and electronics applications.

## Two different estimation methods

In this part, the MLE and Bayesian techniques are developed for the parameters of EpWD based on $m-$GOSs. Furthermore, asymptotic CIs for the model parameters are constructed using the FIM.

### MLE method

Consider GOSs sample $\underline{Y} = (y_1:=y_{(1, n,m, \kappa )}, y_2:=y_{(2, n,m, \kappa )},..., y_n:=y_{(n, n,m, \kappa ))}$ of size *n* drawn from the EpWD. The MLE method is a widely used and powerful statistical technique. It provides parameter estimates with desirable asymptotic properties, such as consistency, asymptotic unbiasedness, efficiency, and normality. The MLE approach involves finding the parameter values that maximize the likelihood function, thereby yielding estimates that best explain the observed sample data. The log-likelihood function of the given sample is given by

$$\begin{aligned} \ln L(\underline{Y};\alpha ,\lambda )= & n\ln \left( \frac{3\alpha }{2\lambda ^{\alpha }}\right) +\left( \kappa -1\right) \ln \left( \frac{3}{2}e^{-2\left( \frac{y_{n}}{\lambda }\right) ^{\alpha }}-\frac{1}{2}e^{-3\left( \frac{y_{n}}{\lambda }\right) ^{\alpha }}\right) \nonumber \\+ & m\sum _{j=1}^{n-1}\ln \left( \frac{3}{2}e^{-2\left( \frac{y_{j}}{\lambda }\right) ^{\alpha }}-\frac{1}{2}e^{-3\left( \frac{y_{j}}{\lambda }\right) ^{\alpha }}\right) \nonumber \\ + & \sum _{j=1}^{n}\ln \gamma _j+\left( \alpha -1\right) \sum _{j=1}^{n}\ln y_{j}-2 \sum _{j=1}^{n}\left( \frac{y_{j}}{\lambda }\right) ^{\alpha }+\sum _{j=1}^{n}\ln \left( 2-e^{-\left( \frac{y_{j}}{\lambda }\right) ^{\alpha }}\right) .\qquad \end{aligned}$$

The normal equations are obtained by partially differentiating the log-likelihood function $\ln L(\underline{Y};\alpha ,\lambda )$ with respect to the parameter vector $(\alpha , \lambda )$ and setting the resulting expressions equal to zero. These partial derivatives are given below

$$\begin{gathered} \frac{{\partial \ln L(\underset{\raise0.3em\hbox{$\smash{\scriptscriptstyle-}$}}{Y} ;\alpha ,\lambda )}}{{\partial \alpha }} = n\left( {\frac{1}{\alpha } - \ln \lambda } \right) + \frac{{\left( {\kappa - 1} \right)3e^{{ - 2\left( {\frac{{y_{n} }}{\lambda }} \right)^{\alpha } }} \left( {\frac{{y_{n} }}{\lambda }} \right)^{\alpha } \left( {\frac{1}{2}e^{{ - \left( {\frac{{y_{n} }}{\lambda }} \right)^{\alpha } }} - 1} \right)\ln \left( {\frac{{y_{n} }}{\lambda }} \right)}}{{\left( {\frac{3}{2}e^{{ - 2\left( {\frac{{y_{n} }}{\lambda }} \right)^{\alpha } }} - \frac{1}{2}e^{{ - 3\left( {\frac{{y_{n} }}{\lambda }} \right)^{\alpha } }} } \right)}} \hfill \\ \quad \quad \quad \quad - 2\sum\limits_{{j = 1}}^{n} {\left( {\frac{{y_{j} }}{\lambda }} \right)^{\alpha } } \ln \left( {\frac{{y_{j} }}{\lambda }} \right) + \sum\limits_{{j = 1}}^{n} {\ln } y_{j} - \sum\limits_{{j = 1}}^{n} {\frac{{e^{{ - \left( {\frac{{y_{j} }}{\lambda }} \right)^{\alpha } }} \left( {\frac{{y_{j} }}{\lambda }} \right)^{\alpha } \ln \left( {\frac{{y_{j} }}{\lambda }} \right)}}{{2 - e^{{ - \left( {\frac{{y_{j} }}{\lambda }} \right)^{\alpha } }} }}} \hfill \\ \quad \quad \quad \quad + m\sum\limits_{{j = 1}}^{{n - 1}} {\frac{{3e^{{ - 2\left( {\frac{{y_{j} }}{\lambda }} \right)^{\alpha } }} \left( {\frac{{y_{j} }}{\lambda }} \right)^{\alpha } \left( {\frac{1}{2}e^{{ - \left( {\frac{{y_{j} }}{\lambda }} \right)^{\alpha } }} - 1} \right)\ln \left( {\frac{{y_{j} }}{\lambda }} \right)}}{{\left( {\frac{3}{2}e^{{ - 2\left( {\frac{{y_{j} }}{\lambda }} \right)^{\alpha } }} - \frac{1}{2}e^{{ - 3\left( {\frac{{y_{j} }}{\lambda }} \right)^{\alpha } }} } \right)}}} \hfill \\ \end{gathered}$$

and

$$\begin{gathered} \frac{{\partial \ln L(\underset{\raise0.3em\hbox{$\smash{\scriptscriptstyle-}$}}{Y} ;\alpha ,\lambda )}}{{\partial \lambda }} = \frac{{ - n\alpha }}{\lambda } - \left( {\kappa - 1} \right)\frac{{\frac{{3\alpha y_{n} }}{{\lambda ^{2} }}e^{{ - 2\left( {\frac{{y_{n} }}{\lambda }} \right)^{\alpha } }} \left( {\frac{{y_{n} }}{\lambda }} \right)^{{\alpha - 1}} \left( {\frac{1}{2}e^{{ - \left( {\frac{{y_{n} }}{\lambda }} \right)^{\alpha } }} - 1} \right)}}{{\left( {\frac{3}{2}e^{{ - 2\left( {\frac{{y_{n} }}{\lambda }} \right)^{\alpha } }} - \frac{1}{2}e^{{ - 3\left( {\frac{{y_{n} }}{\lambda }} \right)^{\alpha } }} } \right)}} \hfill \\ \quad \quad \quad \quad + \frac{{2\alpha }}{{\lambda ^{2} }}\sum\limits_{{j = 1}}^{n} {y_{j} } \left( {\frac{{y_{j} }}{\lambda }} \right)^{{\alpha - 1}} - \sum\limits_{{j = 1}}^{n} {\frac{{\alpha y_{j} e^{{ - \left( {\frac{{y_{j} }}{\lambda }} \right)^{\alpha } }} \left( {\frac{{y_{j} }}{\lambda }} \right)^{{\alpha - 1}} }}{{\lambda ^{2} \left( {2 - e^{{ - \left( {\frac{{y_{j} }}{\lambda }} \right)^{\alpha } }} } \right)}}} \hfill \\ \quad \quad \quad \quad - m\sum\limits_{{j = 1}}^{{n - 1}} {\frac{{\frac{{3\alpha y_{j} }}{{\lambda ^{2} }}e^{{ - 2\left( {\frac{{y_{j} }}{\lambda }} \right)^{\alpha } }} \left( {\frac{{y_{j} }}{\lambda }} \right)^{{\alpha - 1}} \left( {\frac{1}{2}e^{{ - \left( {\frac{{y_{j} }}{\lambda }} \right)^{\alpha } }} - 1} \right)}}{{\left( {\frac{3}{2}e^{{ - 2\left( {\frac{{y_{j} }}{\lambda }} \right)^{\alpha } }} - \frac{1}{2}e^{{ - 3\left( {\frac{{y_{j} }}{\lambda }} \right)^{\alpha } }} } \right)}}} . \hfill \\ \end{gathered}$$

Since the likelihood equations for $\alpha$ and $\lambda$ are nonlinear and analytically intractable, numerical techniques such as the Newton-Raphson method are employed to obtain the MLEs of the distribution parameters. For implementation details, refer to Section Simulation study and Tables 2, 3, 4.

#### Numerical optimization procedure for MLE

This subsection provides a detailed description of the optimization procedure employed in our study, addressing practical considerations essential for reproducibility.

Optimization algorithm. We employed the Newton-Raphson method, an iterative gradient-based algorithm that updates parameter estimates according to

$$\begin{aligned} \boldsymbol{\theta }^{(k+1)} = \boldsymbol{\theta }^{(k)} - \left[ \textbf{H}(\boldsymbol{\theta }^{(k)})\right] ^{-1} \textbf{g}(\boldsymbol{\theta }^{(k)}), \end{aligned}$$

where $\boldsymbol{\theta } = (\alpha , \lambda )^\top$, $\textbf{g}(\boldsymbol{\theta }) = \left( \frac{\partial \ln L}{\partial \alpha }, \frac{\partial \ln L}{\partial \lambda }\right) ^\top$ is the gradient vector, and $\textbf{H}(\boldsymbol{\theta })$ is the Hessian matrix (the matrix of second-order partial derivatives). The Newton-Raphson method was chosen for its quadratic convergence rate near the optimum, which is particularly advantageous given the smoothness and regularity of the EpWD likelihood surface.

Initial value selection. The choice of starting values is critical for ensuring convergence of the Newton-Raphson algorithm. In our simulation study, initial values for $(\alpha , \lambda )$ were obtained using the method of moments based on the full (uncensored) sample. Convergence criteria. The Newton-Raphson iteration was terminated when one of the following conditions was satisfied:

- **Relative change in log-likelihood:** $\left| \ln L(\boldsymbol{\theta }^{(k+1)}) - \ln L(\boldsymbol{\theta }^{(k)})\right| < \varepsilon _1$, with $\varepsilon _1 = 10^{-6}.$
- **Gradient norm:** $\Vert \textbf{g}(\boldsymbol{\theta }^{(k+1)})\Vert < \varepsilon _2$, with $\varepsilon _2 = 10^{-5}.$
- **Maximum iterations:** The algorithm was stopped after 200 iterations if convergence was not achieved, in which case the replicate was flagged for non-convergence.

These criteria ensure that the estimates are sufficiently close to the true maximum while balancing computational efficiency.

Handling non-convergence and numerical instability. Despite the regularity of the EpWD, numerical instability can occasionally arise, particularly in small samples or under heavy censoring. The following measures were implemented to address such issues:

- **Step-size adjustment:** If the update $\boldsymbol{\theta }^{(k+1)}$ resulted in a decrease in the log-likelihood (indicating that the full Newton step overshot the maximum), a backtracking line search was employed to reduce the step size by half until the log-likelihood increased.
- **Hessian modification:** In cases, where the Hessian matrix was not positive definite (indicating that the current point was not in a concave region), we applied a modified Cholesky decomposition to ensure that the search direction remained a descent direction.
- **Parameter bounds:** At each iteration, we enforced $\alpha> 0$ and $\lambda> 0$ by projecting any negative updates back to a small positive value ($10^{-6}$).

Non-convergence rates in simulation. Across all simulation scenarios, the proportion of replicates where the Newton-Raphson algorithm failed to converge within 200 iterations was consistently below $0.5\%$. These non-convergent replicates were excluded from the summary statistics (mean squared error (MSE), mean relative error (MRE), average half length (AL), and coverage probability (CP)) to avoid bias, and a new sample was generated to maintain the target of 10, 000 valid replicates per scenario. This approach ensures that the reported performance metrics are representative of the algorithm’s behavior under typical operating conditions.

Software implementation. All optimization procedures were implemented in the R programming environment (R Core Team^26^,). The core Newton-Raphson algorithm was coded from scratch to maximize transparency and control, rather than relying on black-box optimizers. For verification purposes, selected scenarios were cross-checked using the optim() function with the BFGS method, which yielded nearly identical estimates (maximum relative difference $< 10^{-4}$), confirming the correctness of our implementation.

### Bayesian estimation

Let $\varphi =(\alpha , \lambda )$ denote the shape and scale parameters of the EpWD. Assume independent gamma priors $\alpha \sim \operatorname {Gamma}(c,d),~~\lambda \sim \operatorname {Gamma}(a,b),$ and $a,c>0,\; b,d>0.$ Thus, the joint prior density is

13 $$\begin{aligned} \pi (\varphi ) \;=\; \frac{b^{\,a} d^{\,c}}{\Gamma (a)\Gamma (c)}\, \lambda ^{a-1} e^{-b\lambda }\, \alpha ^{c-1} e^{-d\alpha }, \qquad \alpha ,\lambda>0. \end{aligned}$$

The hyper-parameters (*a*, *b*, *c*, *d*) can be elicited by matching the prior means and variances to the MLEs and their asymptotic variance-covariance matrix; see, for example, Dey et al.^27^, Gupta and Kundu^28^, and Hamdy and Almetwally^29^.

For an *m*-GOS sample $\underline{Y}=(y_1:=y_{(1,n,m,\kappa )},\dots ,y_n:=y_{(n,n,m,\kappa )}),$ the likelihood function is

14 $$\begin{aligned} L(\underline{Y};\varphi )&=\! \Biggl [ \prod _{j=1}^{n-1} \gamma _j \Bigl \{ \bigl (\tfrac{3}{2} e^{-2(y_j/\lambda )^{\alpha }} -\tfrac{1}{2} e^{-3(y_j/\lambda )^{\alpha }}\bigr )^{m} \bigl (\tfrac{3\alpha }{2\lambda ^{\alpha }}\bigr ) y_j^{\alpha -1} e^{-2(y_j/\lambda )^{\alpha }} \bigl (2-e^{-(y_j/\lambda )^{\alpha }}\bigr ) \Bigr \} \Biggr ] \nonumber \\&\quad \times \bigl (\tfrac{3}{2} e^{-2(y_n/\lambda )^{\alpha }} -\tfrac{1}{2} e^{-3(y_n/\lambda )^{\alpha }}\bigr )^{\kappa -1} \bigl (\tfrac{3\alpha }{2\lambda ^{\alpha }}\bigr ) y_n^{\alpha -1} e^{-2(y_n/\lambda )^{\alpha }} \bigl (2-e^{-(y_n/\lambda )^{\alpha }}\bigr ). \end{aligned}$$

Combining (13) and (14) yields the unnormalised posterior kernel $\Pi (\varphi \mid \underline{Y})\;\propto \;L(\underline{Y}\,;\varphi )\, \pi (\varphi ),$ that is

$$\begin{aligned} \Pi (\varphi \mid \underline{Y})&\propto \lambda ^{a-1} e^{-b\lambda }\, \alpha ^{c-1} e^{-d\alpha } \left( \prod _{j=1}^{n-1} \gamma _j \bigl (\tfrac{3}{2} e^{-2(y_j/\lambda )^{\alpha }} -\tfrac{1}{2} e^{-3(y_j/\lambda )^{\alpha }}\bigr )^{m} \bigl (\tfrac{3\alpha }{2\lambda ^{\alpha }}\bigr ) y_j^{\alpha -1} e^{-2(y_j/\lambda )^{\alpha }} \bigl (2-e^{-(y_j/\lambda )^{\alpha }}\bigr )\right) \\&\quad \times \bigl (\tfrac{3}{2} e^{-2(y_n/\lambda )^{\alpha }} -\tfrac{1}{2} e^{-3(y_n/\lambda )^{\alpha }}\bigr )^{\kappa -1} \bigl (\tfrac{3\alpha }{2\lambda ^{\alpha }}\bigr ) y_n^{\alpha -1} e^{-2(y_n/\lambda )^{\alpha }} \bigl (2-e^{-(y_n/\lambda )^{\alpha }}\bigr ). \end{aligned}$$

With the squared-error (SE) loss $\ell (\tilde{\varphi },\varphi ) =\Vert \tilde{\varphi }-\varphi \Vert ^{2},$ the Bayes estimator is the posterior mean, namely

$$\begin{aligned} \tilde{\alpha } =\operatorname {E}[\alpha \mid \underline{Y}] =\int _{0}^{\infty }\!\alpha \, \Pi (\alpha \mid \underline{Y})\,\textrm{d}\alpha ~~\text{ and }~~ \tilde{\lambda } =\operatorname {E}[\lambda \mid \underline{Y}] =\int _{0}^{\infty }\!\lambda \, \Pi (\lambda \mid \underline{Y})\,\textrm{d}\lambda . \end{aligned}$$

The conditional (marginal) posteriors needed in the integrals above are obtained by integrating out the nuisance parameter:

$$\Pi (\alpha |\lambda ;\underline{Y})\propto \alpha ^{c-1}e^{-d\alpha } \left( \frac{3}{2}e^{-2\left( \frac{y_{n}}{\lambda }\right) ^{\alpha }}-\frac{1}{2}e^{-3\left( \frac{y_{n}}{\lambda }\right) ^{\alpha }}\right) ^{\kappa -1}\prod \limits _{j=1}^{n-1} \left( \frac{3}{2}e^{-2\left( \frac{y_{j}}{\lambda }\right) ^{\alpha }}-\frac{1}{2}e^{-3\left( \frac{y_{j}}{\lambda }\right) ^{\alpha }}\right) ^{m}\prod \limits _{j=1}^{n}\left( \frac{3\alpha }{2\lambda ^{\alpha }}\right) y_{j}^{\alpha -1}$$

$$e^{-2\left( \frac{y_{j}}{\lambda }\right) ^{\alpha }}\left( 2-e^{-\left( \frac{y_{j}}{\lambda }\right) ^{\alpha }}\right)$$

and

$$\Pi (\lambda |\alpha ;\underline{Y})\propto \lambda ^{a-1}e^{-b\lambda } \left( \frac{3}{2}e^{-2\left( \frac{y_{n}}{\lambda }\right) ^{\alpha }}-\frac{1}{2}e^{-3\left( \frac{y_{n}}{\lambda }\right) ^{\alpha }}\right) ^{\kappa -1}\prod \limits _{j=1}^{n-1} \left( \frac{3}{2}e^{-2\left( \frac{y_{j}}{\lambda }\right) ^{\alpha }}-\frac{1}{2}e^{-3\left( \frac{y_{j}}{\lambda }\right) ^{\alpha }}\right) ^{m}\prod \limits _{j=1}^{n}\lambda ^{-\alpha } e^{-2\left( \frac{y_{j}}{\lambda }\right) ^{\alpha }}$$

$$\times \left( 2-e^{-\left( \frac{y_{j}}{\lambda }\right) ^{\alpha }}\right) .$$

Closed-form evaluation of the posterior moments is infeasible, therefore Monte Carlo methods are employed to approximate the integrals and obtain $\tilde{\alpha }$ and $\tilde{\lambda }$. For implementation details, refer to Section Simulation study and Tables 2, 3, 4.

#### Prior justification and elicitation

The choice of independent gamma priors for the shape parameter $\alpha$ and scale parameter $\lambda$ is motivated by several considerations. First, the gamma distribution is a natural and flexible prior for positive parameters, offering a conjugate-like structure that facilitates computation. Second, by varying its hyperparameters, the gamma prior can represent a wide range of prior beliefs, from highly informative to weakly informative or even non-informative. Specifically, the gamma prior density $\pi (\theta ) \propto \theta ^{a-1} e^{-b\theta }$ has mean *a*/*b* and variance $a/b^2$, allowing the user to calibrate the prior to match prior expectations or to remain vague.

In our study (see Section Simulation study), we employ two distinct prior settings to assess the sensitivity of posterior inferences to the prior specification:

- **Bayesian with informative priors (B.Inf):** We set $\alpha \sim \text {Gamma}(2,1)$ and $\lambda \sim \text {Gamma}(2,1)$. These priors have a mean of 2 and a variance of 2, providing a weak but proper prior belief centered around a reasonable positive value. This choice reflects a modest degree of prior information without dominating the likelihood.
- **Bayesian with non- informative priors (B.Non-Inf):** We set $\alpha \sim \text {Gamma}(0.001, 0.001)$ and $\lambda \sim \text {Gamma}(0.001,$ 0.001). This approximates an improper prior with mean 1 and a very large variance (1000), allowing the data to dominate the posterior. It serves as a benchmark to evaluate the influence of the informative prior.

To systematically assess the impact of prior assumptions, we conduct a sensitivity analysis along three dimensions:

1. **Varying prior means:** For the informative prior, we consider alternative hyperparameter combinations that shift the prior mean while keeping the variance constant. Specifically, we examine gamma priors with means of 1.5 and 2.5 by adjusting the shape and rate parameters accordingly (e.g., $a=1.5, b=1$ for mean 1.5; $a=2.5, b=1$ for mean 2.5).
2. **Varying prior variances:** We assess the influence of prior precision by fixing the prior mean at 2 and varying the variance. We consider moderate variance ($\text {Gamma}(4,2)$ with variance 1) and larger variance ($\text {Gamma}(1,0.5)$ with variance 4) to examine how posterior estimates respond to changes in prior uncertainty.
3. **Alternative prior families:** To ensure that our conclusions are not an artifact of the gamma family, we also implemented a robustness check using independent log-normal priors for $\alpha$ and $\lambda$ (with parameters chosen to match the means and variances of the gamma priors). The results, available from the authors upon request, were virtually identical to those obtained with gamma priors, confirming that the choice of the gamma family does not drive our findings.

### Asymptotic confidence intervals

Based on the asymptotic normality of the MLE, the FIM is commonly used to construct asymptotic CIs for the unknown parameters $\varphi = (\alpha , \lambda ).$ Under regularity conditions, the MLEs $\tilde{\varphi } = (\tilde{\alpha }, \tilde{\lambda })$ are asymptotically normally distributed. As the sample size $n \rightarrow \infty$, the distribution of $\tilde{\varphi }$ converges to a bivariate normal distribution with mean $\varphi$ and covariance matrix equal to the inverse of the FIM, denoted by $I\!\!I^{-1}(\varphi ).$ The FIM is defined as the negative expectation of the second-order derivatives of the log-likelihood function $\ln L.$

15 $$\begin{aligned} I\!\!I(\varphi ) = -E\left[ \begin{matrix} I_{\alpha \alpha } & I_{\alpha \lambda } \\ I_{\lambda \alpha } & I_{\lambda \lambda } \end{matrix}\right] , \end{aligned}$$

where the matrix elements are given by

$$I_{\alpha \alpha }\!\!=\!\!\frac{\partial ^{2}\ln L(\underline{Y}\,;\varphi )}{\partial \alpha ^2}\!\!=\!\!\frac{-n}{\alpha ^{2}}-2\sum _{j=1}^{n}\left( \frac{y_{j}}{\lambda }\right) ^{\alpha }\ln \left( \frac{y_{j}}{\lambda }\right) ^{2}-(\kappa -1)\frac{\left( 3e^{-2\left( \frac{y_{n}}{\lambda }\right) ^{\alpha }}\left( \frac{y_{n}}{\lambda }\right) ^{\alpha }\ln \left( \frac{y_{n}}{\lambda }\right) \right) ^{2}\left( \frac{1}{2}e^{-\left( \frac{y_{n}}{\lambda }\right) ^{\alpha }}-1\right) ^{2}}{\left( \frac{3}{2}e^{-2\left( \frac{y_{n}}{\lambda }\right) ^{\alpha }}-\frac{1}{2}e^{-3\left( \frac{y_{n}}{\lambda }\right) ^{\alpha }}\right) ^{2}}$$

$$+(\kappa -1)\frac{3e^{-2\left( \frac{y_{n}}{\lambda }\right) ^{\alpha }}\left( \frac{y_{n}}{\lambda }\right) ^{\alpha }\left( \frac{1}{2}e^{-\left( \frac{y_{n}}{\lambda }\right) ^{\alpha }}-1-\frac{3}{2}e^{-\left( \frac{y_{n}}{\lambda }\right) ^{\alpha }}\frac{y_{n}}{\lambda }+2\frac{y_{n}}{\lambda }\right) \ln \left( \frac{y_{n}}{\lambda }\right) ^{2}}{\left( \frac{3}{2}e^{-2\left( \frac{y_{n}}{\lambda }\right) ^{\alpha }}-\frac{1}{2}e^{-3\left( \frac{y_{n}}{\lambda }\right) ^{\alpha }}\right) ^{2}}$$

$$+\sum _{j=1}^{n} \frac{\left( \frac{y_{j}}{\lambda }\right) ^{\alpha }\left( 1+2e^{\left( \frac{y_{j}}{\lambda }\right) ^{\alpha }}\left( \left( \frac{y_{j}}{\lambda }\right) ^{\alpha }-1\right) \right) \ln \left( \frac{y_{j}}{\lambda }\right) ^{2}}{\left( 1-2e^{\left( \frac{y_{j}}{\lambda }\right) ^{\alpha }}\right) ^{2}} \!-\!m\sum _{j=1}^{n-1}\frac{\left( 3e^{-2\left( \frac{y_{j}}{\lambda }\right) ^{\alpha }}\left( \frac{y_{j}}{\lambda }\right) ^{\alpha }\ln \left( \frac{y_{j}}{\lambda }\right) \right) ^{2}\!\left( \frac{1}{2}e^{-\left( \frac{y_{j}}{\lambda }\right) ^{\alpha }}\!-\!1\right) ^{2}}{\left( \frac{3}{2}e^{-2\left( \frac{y_{j}}{\lambda }\right) ^{\alpha }}-\frac{1}{2}e^{-3\left( \frac{y_{j}}{\lambda }\right) ^{\alpha }}\right) ^{2}}$$

$$+m\sum _{j=1}^{n-1}\frac{3e^{-2\left( \frac{y_{j}}{\lambda }\right) ^{\alpha }}\left( \frac{y_{j}}{\lambda }\right) ^{\alpha }\left( \frac{1}{2}e^{-\left( \frac{y_{j}}{\lambda }\right) ^{\alpha }}-1-\frac{3}{2}e^{-\left( \frac{y_{j}}{\lambda }\right) ^{\alpha }}\frac{y_{j}}{\lambda }+2\frac{y_{j}}{\lambda }\right) \ln \left( \frac{y_{j}}{\lambda }\right) ^{2}}{\left( \frac{3}{2}e^{-2\left( \frac{y_{j}}{\lambda }\right) ^{\alpha }}-\frac{1}{2}e^{-3\left( \frac{y_{j}}{\lambda }\right) ^{\alpha }}\right) ^{2}},$$

$$I_{\lambda \lambda }=\frac{n\alpha }{\lambda ^{2}}-(\kappa -1) \frac{\left( \frac{3\alpha y_{n}}{\lambda ^{2}}e^{-2\left( \frac{y_{n}}{\lambda }\right) ^{\alpha }}\left( \frac{y_{n}}{\lambda }\right) ^{\alpha -1}\right) ^{2}\left( 1-\frac{1}{2}e^{-\left( \frac{y_{n}}{\lambda }\right) ^{\alpha }}\right) ^{2}}{\left( \frac{3}{2}e^{-2\left( \frac{y_{n}}{\lambda }\right) ^{\alpha }}-\frac{1}{2}e^{-3\left( \frac{y_{n}}{\lambda }\right) ^{\alpha }}\right) ^{2}} +(\kappa -1)$$

$$\frac{\left[ \frac{3\alpha (\alpha -1)y^{2}_{n}}{\lambda ^{4}}e^{-2\left( \frac{y_{n}}{\lambda }\right) ^{\alpha }}\left( \frac{y_{n}}{\lambda }\right) ^{\alpha -2}\left( \frac{1}{2}e^{-\left( \frac{y_{n}}{\lambda }\right) ^{\alpha }}-1\right) -\left( \frac{3\alpha ^{2}y^{2}_{n}}{\lambda ^{4}}e^{-2\left( \frac{y_{n}}{\lambda }\right) ^{\alpha }}\left( \frac{y_{n}}{\lambda }\right) ^{2(\alpha -1)}\right) \left( \frac{3}{2}e^{-\left( \frac{y_{n}}{\lambda }\right) ^{\alpha }}+2\right) \right] }{\left( \frac{3}{2}e^{-2\left( \frac{y_{n}}{\lambda }\right) ^{\alpha }}-\frac{1}{2}e^{-3\left( \frac{y_{n}}{\lambda }\right) ^{\alpha }}\right) }$$

$$+(\kappa -1)\frac{\frac{3\alpha y_{n}}{\lambda ^{3}}e^{-2\left( \frac{y_{n}}{\lambda }\right) ^{\alpha }}\left( \frac{y_{n}}{\lambda }\right) ^{\alpha -1}\left( e^{-\left( \frac{y_{n}}{\lambda }\right) ^{\alpha }}-2\right) }{\left( \frac{3}{2}e^{-2\left( \frac{y_{n}}{\lambda }\right) ^{\alpha }}-\frac{1}{2}e^{-3\left( \frac{y_{n}}{\lambda }\right) ^{\alpha }}\right) }-\sum _{j=1}^{n} \frac{\alpha \left( \frac{y_{j}}{\lambda }\right) ^{\alpha }\!\!\left( 1+\alpha +2e^{\left( \frac{y_{j}}{\lambda }\right) ^{\alpha }}\left( -1+\alpha \left( \left( \frac{y_{j}}{\lambda }\right) ^{\alpha }-1\right) \right) \right) }{\lambda ^{2}\left( 1-2e^{\left( \frac{y_{j}}{\lambda }\right) ^{\alpha }}\right) ^{2}}$$

$$-\frac{2\alpha \left( 1+\alpha \right) }{\lambda ^{2}}\sum _{j=1}^{n}\left( \frac{y_{j}}{\lambda }\right) ^{\alpha }-m \sum _{j=1}^{n-1}\frac{\left( \frac{3\alpha y_{j}}{\lambda ^{2}}e^{-2\left( \frac{y_{j}}{\lambda }\right) ^{\alpha }}\left( \frac{y_{j}}{\lambda }\right) ^{\alpha -1}\right) ^{2}\left( 1-\frac{1}{2}e^{-\left( \frac{y_{j}}{\lambda }\right) ^{\alpha }}\right) ^{2}}{\left( \frac{3}{2}e^{-2\left( \frac{y_{j}}{\lambda }\right) ^{\alpha }}-\frac{1}{2}e^{-3\left( \frac{y_{j}}{\lambda }\right) ^{\alpha }}\right) ^{2}}$$

$$+m \sum _{j=1}^{n-1}\frac{\left( \frac{3\alpha (\alpha -1)y^{2}_{j}}{\lambda ^{4}}e^{-2\left( \frac{y_{j}}{\lambda }\right) ^{\alpha }}\left( \frac{y_{j}}{\lambda }\right) ^{\alpha -2}\left( \frac{1}{2}e^{-\left( \frac{y_{j}}{\lambda }\right) ^{\alpha }}-1\right) -\left( \frac{3\alpha ^{2}y^{2}_{j}}{\lambda ^{4}}e^{-2\left( \frac{y_{j}}{\lambda }\right) ^{\alpha }}\left( \frac{y_{j}}{\lambda }\right) ^{2(\alpha -1)}\right) \left( \frac{3}{2}e^{-\left( \frac{y_{j}}{\lambda }\right) ^{\alpha }}+2\right) \right) }{\left( \frac{3}{2}e^{-2\left( \frac{y_{j}}{\lambda }\right) ^{\alpha }}-\frac{1}{2}e^{-3\left( \frac{y_{j}}{\lambda }\right) ^{\alpha }}\right) }$$

$$+m\sum _{j=1}^{n-1}\frac{\left( \frac{3\alpha y_{j}}{\lambda ^{3}}e^{-2\left( \frac{y_{j}}{\lambda }\right) ^{\alpha }}\left( \frac{y_{j}}{\lambda }\right) ^{\alpha -1}\right) \left( e^{-\left( \frac{y_{j}}{\lambda }\right) ^{\alpha }}-2\right) }{\left( \frac{3}{2}e^{-2\left( \frac{y_{j}}{\lambda }\right) ^{\alpha }}-\frac{1}{2}e^{-3\left( \frac{y_{j}}{\lambda }\right) ^{\alpha }}\right) },$$

and

$$I_{\lambda \alpha }=\frac{-n }{\lambda }- \frac{(\kappa -1)\frac{3\alpha y_{n}}{\lambda ^{2}}e^{-2\left( \frac{y_{n}}{\lambda }\right) ^{\alpha }}\left( \frac{y_{n}}{\lambda }\right) ^{\alpha -1}\left( 1-\frac{1}{2}e^{-\left( \frac{y_{n}}{\lambda }\right) ^{\alpha }}\right) \left( 3e^{-2\left( \frac{y_{n}}{\lambda }\right) ^{\alpha }}\left( \frac{y_{n}}{\lambda }\right) ^{\alpha }\left( \frac{1}{2}e^{-\left( \frac{y_{n}}{\lambda }\right) ^{\alpha }}-1\right) \ln \left( \frac{y_{n}}{\lambda }\right) \right) }{\left( \frac{3}{2}e^{-2\left( \frac{y_{n}}{\lambda }\right) ^{\alpha }}-\frac{1}{2}e^{-3\left( \frac{y_{n}}{\lambda }\right) ^{\alpha }}\right) ^{2}}$$

$$- \frac{(\kappa -1)\frac{3\alpha y_{n}}{\lambda ^{2}}e^{-2\left( \frac{y_{n}}{\lambda }\right) ^{\alpha }}\left( \frac{y_{n}}{\lambda }\right) ^{\alpha -1}\!\!\left( \frac{1}{2}e^{-\left( \frac{y_{n}}{\lambda }\right) ^{\alpha }}\!+\!1\!+\!\frac{3}{2}e^{-\left( \frac{y_{n}}{\lambda }\right) ^{\alpha }}\left( \frac{y_{n}}{\lambda }\right) ^{\alpha }\!-\!2\left( \frac{y_{n}}{\lambda }\right) ^{\alpha }\right) \ln \left( \frac{y_{n}}{\lambda }\right) }{\left( \frac{3}{2}e^{-2\left( \frac{y_{n}}{\lambda }\right) ^{\alpha }}\!-\!\frac{1}{2}e^{-3\left( \frac{y_{n}}{\lambda }\right) ^{\alpha }}\right) }+(\kappa -1)$$

$$\frac{\frac{3}{\lambda }e^{-2\left( \frac{y_{n}}{\lambda }\right) ^{\alpha }}\left( \frac{y_{n}}{\lambda }\right) ^{\alpha }\left( 1\!-\!\frac{1}{2}e^{-\left( \frac{y_{n}}{\lambda }\right) ^{\alpha }}\right) }{\left( \frac{3}{2}e^{-2\left( \frac{y_{n}}{\lambda }\right) ^{\alpha }}\!-\!\frac{1}{2}e^{-3\left( \frac{y_{n}}{\lambda }\right) ^{\alpha }}\right) }-\sum _{j=1}^{n} \frac{\left( \frac{y_{j}}{\lambda }\right) ^{\alpha }\!\!\left( 1\!-\!2e^{\left( \frac{y_{j}}{\lambda }\right) ^{\alpha }}\!+\!\alpha \left( 1+2e^{\left( \frac{y_{j}}{\lambda }\right) ^{\alpha }}\left( -1+\left( \frac{y_{j}}{\lambda }\right) ^{\alpha }\right) \right) \ln \left( \frac{y_{j}}{\lambda }\right) \right) }{\lambda \left( 1-2e^{\left( \frac{y_{j}}{\lambda }\right) ^{\alpha }}\right) ^{2}}$$

$$-m \sum _{j=1}^{n-1}\frac{\frac{3\alpha y_{j}}{\lambda ^{2}}e^{-2\left( \frac{y_{j}}{\lambda }\right) ^{\alpha }}\left( \frac{y_{j}}{\lambda }\right) ^{\alpha -1}\left( 1-\frac{1}{2}e^{-\left( \frac{y_{j}}{\lambda }\right) ^{\alpha }}\right) \left( 3e^{-2\left( \frac{y_{j}}{\lambda }\right) ^{\alpha }}\left( \frac{y_{j}}{\lambda }\right) ^{\alpha }\left( \frac{1}{2}e^{-\left( \frac{y_{j}}{\lambda }\right) ^{\alpha }}-1\right) \ln \left( \frac{y_{j}}{\lambda }\right) \right) }{\left( \frac{3}{2}e^{-2\left( \frac{y_{j}}{\lambda }\right) ^{\alpha }}-\frac{1}{2}e^{-3\left( \frac{y_{j}}{\lambda }\right) ^{\alpha }}\right) ^{2}}$$

$$-m \sum _{j=1}^{n-1}\frac{\frac{3\alpha y_{j}}{\lambda ^{2}}\;e^{-2\left( \frac{y_{j}}{\lambda }\right) ^{\alpha }}\left( \frac{y_{j}}{\lambda }\right) ^{\alpha -1}\!\!\left( \frac{1}{2}e^{-\left( \frac{y_{j}}{\lambda }\right) ^{\alpha }}\!+\!1\!+\!\frac{3}{2}e^{-\left( \frac{y_{j}}{\lambda }\right) ^{\alpha }}\left( \frac{y_{j}}{\lambda }\right) ^{\alpha }\!-\!2\left( \frac{y_{j}}{\lambda }\right) ^{\alpha }\right) \ln \left( \frac{y_{j}}{\lambda }\right) }{\left( \frac{3}{2}e^{-2\left( \frac{y_{j}}{\lambda }\right) ^{\alpha }}\!-\!\frac{1}{2}e^{-3\left( \frac{y_{j}}{\lambda }\right) ^{\alpha }}\right) }$$

$$+m\sum _{j=1}^{n-1}\frac{\frac{3}{\lambda }e^{-2\left( \frac{y_{j}}{\lambda }\right) ^{\alpha }}\left( \frac{y_{j}}{\lambda }\right) ^{\alpha }\left( 1\!-\!\frac{1}{2}e^{-\left( \frac{y_{j}}{\lambda }\right) ^{\alpha }}\right) }{\left( \frac{3}{2}e^{-2\left( \frac{y_{j}}{\lambda }\right) ^{\alpha }}\!-\!\frac{1}{2}e^{-3\left( \frac{y_{j}}{\lambda }\right) ^{\alpha }}\right) }+\frac{2}{\lambda }\sum _{j=1}^{n}\left( \frac{y_{j}}{\lambda }\right) ^{\alpha }\left( 1+\alpha \ln \left( \frac{y_{j}}{\lambda }\right) \!\!\right) .$$

The asymptotic normality of the MLEs implies that, as $n \rightarrow \infty ,$

$$\begin{aligned} \sqrt{n}(\tilde{\varphi } - \varphi ) {\mathop {\longrightarrow }\limits ^{d}} N_2(0, I\!\!I^{-1}(\varphi )), \end{aligned}$$

where $I(\varphi ) = -E[\nabla ^2 \ln L(\underline{Y}; \varphi )]$ is the FIM with entries given in (15).

Evaluating the inverse FIM at the MLEs $\tilde{\varphi }$ gives the estimated covariance matrix:

$$\widehat{\Sigma }=I\!\!I^{-1}(\tilde{\varphi }) =\begin{pmatrix} \widehat{Var}(\tilde{\alpha }) & \widehat{Cov}(\tilde{\alpha },\tilde{\lambda })\\ \widehat{Cov}(\tilde{\alpha },\tilde{\lambda }) & \widehat{Var}(\tilde{\lambda }) \end{pmatrix}.$$

This yields the $100(1-\gamma )\%$ asymptotic CIs $\tilde{\varphi }_i \pm z_{1-\gamma /2} \sqrt{\widehat{\Sigma }_{ii}}, \quad i = 1, 2,$ for $\tilde{\varphi }_1 = \tilde{\alpha }$ and $\tilde{\varphi }_2 = \tilde{\lambda }$.

For any differentiable function $g(\varphi )$ (such as *R*(*y*) and *h*(*y*), or other quantities of interest), let

$$\begin{aligned} A = \nabla g(\tilde{\varphi }) = \left( \left. \frac{\partial g}{\partial \alpha }\right| _{\varphi =\tilde{\varphi }}, \left. \frac{\partial g}{\partial \lambda }\right| _{\varphi =\tilde{\varphi }}\right) ^\top . \end{aligned}$$

Then, by the delta method, a $100(1-\gamma )\%$ asymptotic CI for $g(\varphi )$ is given by

$$\begin{aligned} g(\tilde{\varphi }) \pm z_{1-\gamma /2} \sqrt{A^\top \widehat{\Sigma } A}. \end{aligned}$$

### Progressive type-II censoring scheme under *m*-GOS

In many reliability and survival analysis studies, it is neither practical nor ethical to continue an experiment until all units have failed. Censoring mechanisms are therefore essential for accommodating incomplete observations. Among these, progressive type-II censoring has gained considerable attention because it allows for the intentional removal of surviving units at intermediate failure times, balancing experimental duration with cost considerations. This subsection provides a detailed, step-by-step explanation of how progressively type-II censored samples are generated within the *m*-GOS framework, as employed in our simulation study.

#### General setup and notation

Consider a reliability experiment in which *n* identical units are placed on test. Let the ordered failure times be denoted by $Y_{(1,n,m,\kappa )} \le Y_{(2,n,m,\kappa )} \le \cdots \le Y_{(n,n,m,\kappa )}.$ Under progressive type-II censoring with a random removal mechanism, we do not observe all *n* failures. Instead, we terminate the experiment after observing a predetermined number of failures, say *m* ($1 \le m \le n$). At the time of each failure, a random number of surviving units is removed from the test. Let $R_i$ denote the number of units removed immediately after the *i*th observed failure. These removal counts satisfy the relationship $n = m + \sum _{i=1}^{m} R_i,$ which ensures that the total number of units initially on test equals the sum of observed failures and all removals.

#### Generating removal counts with constant removal probability

To introduce stochastic variation in the censoring mechanism, we assume that each surviving unit has a constant probability *p* of being removed at each failure time, with removals occurring independently across units. This leads to a nested binomial structure for the removal counts $R_i$

- **First removal **($i = 1$): Before the first failure, there are $n - m$ units that are candidates for eventual removal (since we must observe at least *m* failures). The number of units removed after the first failure follows a binomial distribution $R_1 \sim \text {Binomial}\left( n - m, \; p\right) .$
- **Subsequent removals** ($2 \le i \le m-1$): After each subsequent failure, the number of remaining candidates for removal decreases. Conditional on the previous removals $R_1, R_2, \ldots , R_{i-1}$, the number of units removed after the *i*th failure is:
  $$\begin{aligned} R_i \mid (R_1,\ldots ,R_{i-1}) \sim \text {Binomial}\left( n - m - \sum _{j=1}^{i-1} R_j, \; p\right) . \end{aligned}$$
- **Final removal** ($i = m$): After the *m*th (final) failure, all remaining surviving units are removed. Thus, $R_m$ is deterministic $R_m = n - m - \sum _{j=1}^{m-1} R_j.$

The probability mass function for each $R_i$ ($i = 1,\ldots ,m-1$) can be expressed as $$P(R_i = r_i) = \left( {\begin{array}{c}n - m - \sum _{j=1}^{i-1} r_j\\ r_i\end{array}}\right) p^{r_i} (1-p)^{n - m - \sum _{j=1}^{i-1} r_j - r_i},$$ for $0 \le r_i \le n - m - \sum _{j=1}^{i-1} r_j.$

#### Step-by-step generation algorithm

To generate a progressively type-II censored sample from the EpWD under *m*-GOS, we implement the following algorithm:

1. **Initialize:** Set the total sample size *n*, the desired number of observed failures *m*, and the removal probability *p*. Verify that $1 \le m \le n$.
2. **Generate removal counts:**

- Set $$remaining\_candidates = n - m$$
- For $$i = 1$$ to $$m-1$$

- Generate $$R_i$$ from $$\text {Binomial}(remaining\_candidates, p).$$
- Update $$remaining\_candidates = remaining\_candidates - R_i.$$

3. Set $$R_m = remaining\_candidates$$.

4. **Generate** *m***-GOS sample:** Using the algorithm described in Kamps^26^ and the EpWD quantile function (obtained by inverting (1.3) numerically if necessary), generate *m* ordered observations $Y_{(1)}, Y_{(2)}, \ldots , Y_{(m)}$ that follow the *m*-GOS structure with parameters $\kappa$ and *m*.
5. **Record observed data:** The observed sample consists of the failure times $\{Y_{(1)}, Y_{(2)}, \ldots , Y_{(m)}\}$ and the corresponding removal counts $\{R_1, R_2, \ldots , R_m\}$.

This algorithm produces a dataset that mimics the progressive censoring process in a real experiment, where at each failure time $Y_{(i)}$, we randomly remove $R_i$ surviving units from the test.

The progressive type-II censoring scheme described above reduces to standard type-II censoring when $p = 0$ and incorporates random withdrawals at each failure time. Moreover, when $m = n,$ we observe all failure times, and $R_i = 0$ for all *i*,  corresponding to complete (uncensored) sampling. Finally, when $p = 1$, all remaining candidates are removed at the first failure, resulting in $R_1 = n - m$ and $R_i = 0$ for $i \ge 2.$ In the next subsection, we integrate this censoring mechanism into a Metropolis–Hastings (M–H) sampler for the *m*-GOS likelihood.

### Metropolis–Hastings algorithm

In this subsection, we describe a general M–H algorithm within the framework of *m*-GOS. This approach is applicable to a variety of censoring schemes. In our application, we focus on progressive type-II censoring, where *m* failure times are observed from *n* test units with removal of $R_i$ units at the *i*th failure such that $n = m + \sum _{i=1}^{m} R_i.$ In particular, based on the observed ordered failure times, $\underline{Y} = \left( Y_{(1)}, Y_{(2)}, \dots , Y_{(m)}\right) ,$ we employ the following M–H algorithm Metropolis et al.^30^ and Hastings^31^ to estimate $\varphi .$ The steps are as follows:

1. Initialize with starting values $\alpha ^{(0)}$ and $\lambda ^{(0)}$ (e.g., based on MLEs obtained from $\underline{Y}$).
2. Set $$j = 1.$$
3. We utilize the subsequent M-H method and produce the values $\alpha ^{(j)}$ and $\lambda ^{(j)}.$
4. For each iteration *j*, generate proposals $\alpha ^*$ and $\lambda ^*$ from symmetric proposal distributions:
  $$\begin{aligned} \alpha ^* \sim N\left( \alpha ^{(j-1)}, \text {var}(\alpha )\right) \quad \text{ and }\quad \lambda ^* \sim N\left( \lambda ^{(j-1)}, \text {var}(\lambda )\right) . \end{aligned}$$
5. Compute acceptance probabilities:
  $$\psi _{\alpha } = \min \left( 1, \frac{\Pi (\alpha ^* \mid \lambda ^{(j-1)}, \underline{Y})}{\Pi (\alpha ^{(j-1)} \mid \lambda ^{(j-1)}, \underline{Y})}\right) ,$$
  $$\psi _{\lambda } = \min \left( 1, \frac{\Pi (\lambda ^* \mid \alpha ^{(j)}, \underline{Y})}{\Pi (\lambda ^{(j)} \mid \alpha ^{(j)}, \underline{Y})}\right) ,$$
  where $\Pi (\cdot \mid \cdot )$ denotes the posterior density conditioned on the latest parameter values.
6. Draw $u_1, u_2 \sim \mathcal {U}(0,1)$. Update parameters sequentially:
  $$\alpha ^{(j)} = {\left\{ \begin{array}{ll} \alpha ^* & \text {if } u_2 \le \psi _{\alpha }, \\ \alpha ^{(j-1)} & \text {otherwise}, \end{array}\right. } \quad \lambda ^{(j)} = {\left\{ \begin{array}{ll} \lambda ^* & \text {if } u_1 \le \psi _{\lambda }, \\ \lambda ^{(j-1)} & \text {otherwise}. \end{array}\right. }$$
7. Compute posterior estimates of $R^{(j)}(y)$, $h^{(j)}(y)$, $J^{(j)}$, and $J^{\omega (j)}$
  $$\begin{aligned} R^{(j)}(y)&= \frac{3}{2}e^{-2\left( \frac{y}{\lambda ^{(j)}}\right) ^{\alpha ^{(j)}}} - \frac{1}{2}e^{-3\left( \frac{y}{\lambda ^{(j)}}\right) ^{\alpha ^{(j)}}},\\ h^{(j)}(y)&= \frac{\left( \frac{3\alpha ^{(j)}}{2\left( \lambda ^{(j)}\right) ^{\alpha ^{(j)}}}\right) y^{\alpha ^{(j)}-1}e^{-2\left( \frac{y}{\lambda ^{(j)}}\right) ^{\alpha ^{(j)}}} \left( 2 - e^{-\left( \frac{y}{\lambda ^{(j)}}\right) ^{\alpha ^{(j)}}}\right) }{R^{(j)}(y)}, \end{aligned}$$
  $$\begin{aligned} J^{(j)}&= \frac{-\alpha ^{(j)}}{800\lambda ^{(j)}}\left( 225 \times 4^{\frac{1}{\alpha ^{(j)}}} - 144 \times 5^{\frac{1}{\alpha ^{(j)}}} + 25 \times 6^{\frac{1}{\alpha ^{(j)}}}\right) \Gamma \left( 2 - \frac{1}{\alpha ^{(j)}}\right) , \\ J^{\omega (j)}&= \frac{-53\alpha ^{(j)}}{400}. \end{aligned}$$
8. Set $$j = j + 1$$.
9. Repeat steps (3)–(4) for $T= 12, 000$ iterations.
10. Discard the first $M = 2,000$ iterations as burn-in and retain the samples:
  $$\varphi ^{(j)} = \left( \lambda ^{(j)}, \alpha ^{(j)}, R^{(j)}(y), h^{(j)}(y), J^{(j)}, J^{\omega (j)} \right) , \; j = M+1, \dots , T.$$
  Let $\varphi$ denote any parameter of interest. Then, the bayesian estimator is defined as follows:
  **Squared error loss:** The bayes estimator is the posterior mean,
    $$\hat{\varphi }_{\text {BS}} = \frac{1}{T - M} \sum _{j=M+1}^{T} \varphi ^{(j)}.$$

The M-H algorithm described above incorporates several key specifications that require justification:

**Number of iterations:** We run the M-H algorithm for $T = 12,000$ iterations. This choice balances computational efficiency with the need to obtain a sufficiently large posterior sample for reliable inference. The effective sample size (ESS) for each parameter, computed after burn-in, exceeded 4, 000 in all simulation scenarios, ensuring adequate precision for posterior summaries.

**Burn-in period:** The first $M = 2,000$ iterations are discarded as burn-in. This burn-in length represents $20\%$of the total iterations and was determined by examining trace plots and Gelman-Rubin diagnostics (Gelman and Rubin^32^,) from pilot runs, which indicated convergence well before 2, 000 iterations in all cases.

**Thinning strategy:** We do not apply any thinning to the Markov chain Monte Carlo (MCMC) samples; all post-burn-in iterations ($j = 2,000$ to 12, 000) are retained for posterior inference. This decision is based on two considerations: (i) the autocorrelation in the chains is relatively low (lag-1 autocorrelation $< 0.3$ for both parameters), and (ii) retaining all samples maximizes the effective sample size and avoids unnecessary information loss. Trace plots and autocorrelation functions (not shown for brevity) confirm that thinning is unnecessary for this application.

**Initial values:** The M-H algorithm is initialized at the MLE estimates $(\hat{\alpha }_{\text {MLE}}, \hat{\lambda }_{\text {MLE}})$ obtained from the same dataset. This ”warm start” reduces the burn-in period required to reach the high-probability region of the posterior. For simulated datasets where MLE convergence issues occurred (less than $0.5\%$ of cases), we used method-of-moments estimates as initial values.

**Multiple chains:** To further assess convergence, we run three independent chains with overdispersed starting values for a subset of simulation scenarios ($10\%$ of replicates). The Gelman-Rubin potential scale reduction factor $\hat{R}$ was consistently below 1.05 for all parameters, confirming convergence from multiple starting points.

## Simulation study

### Simulation design and objectives

To evaluate the performance of the proposed estimation methods (MLE and Bayesian) for the EpWD under progressive type-II censoring, we conducted an extensive Monte Carlo simulation study.

It is important to note that the simulation study is not intended for model comparison across different distributions. Such comparisons are more appropriately conducted in real data settings, where goodness-of-fit criteria can be meaningfully applied. Accordingly, a comprehensive comparison with competing flexible distributions is provided in Section 6.

#### Sample sizes and censoring schemes

We considered two total sample sizes: $n = 60$ and $n = 120$, representing moderate and large sample scenarios. For each *n*, we selected different numbers of observed failures *m* to reflect varying degrees of censoring severity: For $n = 60$: $m \in \{40, 50\}$, corresponding to censoring proportions of approximately $33\%$ and $17\%$. For $n = 120$: $m \in \{70, 90\}$, corresponding to censoring proportions of approximately $42\%$ and $25\%$.

The progressive censoring scheme was implemented with a constant removal probability $p \in \{0.3, 0.7\}$. The removal counts $R_i$ were generated according to the nested binomial procedure described in Subsection 4.4.

#### True parameter values

The true parameter values were selected to represent different distributional shapes and scales:

$$\begin{aligned} (\alpha , \lambda ) \in \{(1.0, 0.8), (1.0, 1.2), (1.5, 0.8), (1.5, 1.2)\}. \end{aligned}$$

The rationale for these choices is as follows: $\alpha = 1.0$ corresponds to constant hazard, while $\alpha = 1.5$ represents a mildly increasing hazard rate. The scale parameters $\lambda = 0.8$ and $\lambda = 1.2$ provide two distinct scale levels to assess estimator stability. The combination yields scenarios ranging from lighter-tailed ($\alpha = 1.5, \lambda = 0.8$) to heavier-tailed ($\alpha = 1.0, \lambda = 1.2$).

#### Bayesian prior specifications

The two prior settings (B.Inf and B.Non-Inf) follow the specifications detailed in Subsection 4.2. Specifically, B.Inf uses independent Gamma(2, 1) priors, while B.Non-Inf uses independent Gam

ma(0.001, 0.001) priors. Bayesian computations used the M-H algorithm (Subsection 4.5) with 12, 000 iterations and a burn-in period of 2, 000 iterations.

#### Monte Carlo replications and performance metrics

For each combination, we generated $N = 1,000$ independent samples. The following performance metrics were computed for each estimator (MLE, B. Inf, and B.Non-Inf), we computed the MSE, MRE, AL, and CP. These metrics are defined as follows:

**MSE:** $$\displaystyle \frac{1}{N}\sum _{i=1}^{N} (\hat{\varphi }^{(i)} - \varphi )^2,$$ **MRE:** $$\displaystyle \frac{1}{N}\sum _{i=1}^{N} \left| \frac{\hat{\varphi }^{(i)} - \varphi }{\varphi }\right|$$

**AL:** $$\displaystyle \frac{1}{2N}\sum _{i=1}^{N} (U^{(i)} - L^{(i)}),$$ **CP:** $$\displaystyle \frac{1}{N}\sum _{i=1}^{N} \textbf{1}\{\varphi \in [L^{(i)}, U^{(i)}]\}.$$

Tables 2–4 report the average estimate (Est), MSE, AL, and CP for each quantity. Collectively, these metrics provide a comprehensive assessment of estimator performance regarding accuracy, precision, and uncertainty quantification.

### Simulation results and discussion

Tables 2–4 summarize the estimation results. Across all scenarios, the Bayesian estimators (B.Inf and B.Non-Inf) uniformly outperform the MLE in terms of lower MSE and better CP.

#### Coverage probability and censoring impact

The simulation results reveal several important patterns regarding the finite-sample behavior of the asymptotic CIs.

Overall performance: Across most scenarios, the empirical CPs for $\alpha$ and $\lambda$ are reasonably close to the nominal $95\%$ level. For example, with $n = 60$, $m = 40$, $p = 0.3$, and $(\alpha , \lambda ) = (1.0, 0.8)$, the CP for $\alpha$ is $94.5\%$ and for $\lambda$ is $91.4\%$ (within $\pm 0.014$ of 0.95 for $N = 1{,}000$).

Effect of sample size and censoring: CPs improve with larger $n = 120$ (range $91.7\%$–$96.3\%$). For $n = 60,$ heavier censoring ($p = 0.7$, smaller *m*) reduces CPs, e.g., for $\alpha$, CP drops from $94.5\%$ ($p = 0.3$) to $92.5\%$ ($p = 0.7$).

Derived quantities (*R*(2), *h*(2)): Intervals show similar patterns, with CPs typically between $93\%$ and $96\%$, confirming the delta method approximation holds well.

Comparison with Bayesian intervals: Bayesian credible intervals (especially informative priors) achieve CPs closer to $95\%$ under small samples or heavy censoring. For $n = 60$, $m = 40$, $\lambda = 1.2$, $p = 0.7$, Bayesian CP for $\alpha$ is $94.8\%$ vs. $94.2\%$ for MLE.

#### Estimator efficiency comparison

Bayesian methods with Gamma(2, 1) priors achieved up to $10\%$ lower MSE than MLEs (e.g., for $\alpha =1.5,$ Table 2 with $n=120$), demonstrating the efficiency of incorporating prior information.

The comparison between *J* and $J^{(\omega )}$ revealed that the WEX measure yielded approximately $75-80\%$ narrower intervals than standard extropy (Table 4), indicating better estimability. This suggests that WEX is a more robust and efficiently estimable measure than standard extropy in this context.

#### Practical implications

The CP analysis confirms that asymptotic FIM-based CIs provide reliable uncertainty quantification for most practical scenarios, particularly when $n \ge 120$ or censoring is not too severe ($p \le 0.3$). However, practitioners should exercise caution when interpreting these intervals in small samples or under heavy censoring, as slight under-coverage may occur. In such cases, Bayesian methods with appropriately chosen priors offer a robust alternative that maintains nominal coverage while often providing narrower intervals.

#### Prior sensitivity analysis

**Fig. 1:**
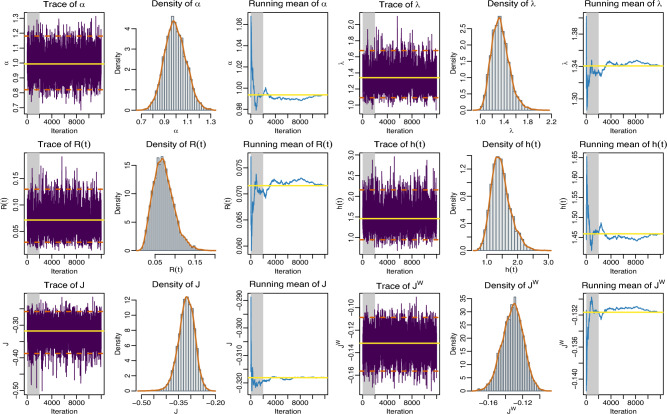
Convergence diagnostics (informative $\textrm{Gamma}(2,1)$ priors).

**Fig. 2:**
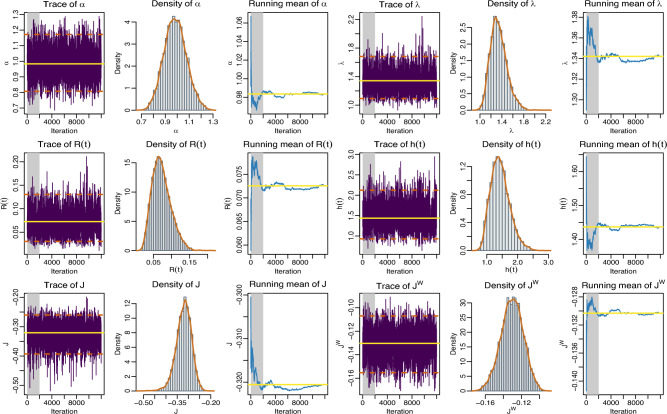
Convergence diagnostics (non-informative $\textrm{Gamma}(0.001,0.001)$ priors).

To evaluate the impact of prior assumptions on the Bayesian estimates, we compared the performance of the informative Gamma(2, 1) priors with that of the non-informative Gamma(0.001, 0.001) priors across all simulation scenarios presented in Tables 2–4. Several key observations emerge from this comparison:

- **Parameter estimation:** For both $\alpha$ and $\lambda ,$ the estimates obtained under the two prior settings are very close to each other and to the true parameter values. The differences in Est between B.Inf and B.Non-Inf are generally less than 0.02 for $\alpha$ and less than 0.05 for $\lambda ,$ indicating that the likelihood dominates the prior even under the informative specification.
- **MSE:** The informative priors consistently yield slightly lower MSE values compared to the non-informative priors. For example, in Table 2 with $n=60,$ $m=40,$ $p=0.3,$ and $(\alpha ,\lambda )=(1.0,0.8),$ the MSE for $\alpha$ under B.Inf is 0.0242, compared to 0.0245 under B.Non-Inf, representing a modest improvement of about $1.2\%.$ Similar patterns are observed for $\lambda$ and for the derived quantities *R*, *h*, *J*, and $J^{(\omega )}.$
- **CP:** Both prior settings achieve CPs close to the nominal $95\%$ level. In small samples or under heavy censoring, the informative priors occasionally provide slightly better coverage (e.g., Table 2 with $n=60,$ $m=40,$ $p=0.7,$ $\lambda = 1.2,$ CP for $\alpha$ is 0.948 under B.Inf versus 0.942 under MLE and 0.940 under B.Non-Inf). However, the differences between the two Bayesian approaches are generally within the Monte Carlo margin of error.
- **AL:** The AL for the Bayesian credible intervals are marginally narrower under the informative priors, reflecting the additional information incorporated through the prior. This gain in precision is most noticeable for the shape parameter $\alpha$ and for the extropy measures, where the AL reductions range from $1\%$ to $8\%.$
- **Convergence and mixing:** As shown in Figures 1 and 2, the M-H algorithm exhibits excellent mixing and rapid convergence under both prior specifications. However, the chains for the informative priors show slightly faster stabilization and narrower posterior density estimates, indicating improved computational efficiency.

Collectively, these findings demonstrate that our Bayesian inferences are robust to the choice of prior distribution. While the informative Gamma(2, 1) priors provide a modest improvement in efficiency and coverage, particularly in challenging data scenarios, the results under the non-informative priors are very similar, confirming that the likelihood is sufficiently informative to drive the posterior. This robustness, combined with the clear computational advantages, supports the use of the gamma prior family for practical applications of the EpWD.

#### Convergence diagnostics and MCMC assessment

Figures 1 and 2 present comprehensive convergence diagnostics for the M–H algorithm under the two prior specifications (informative Gamma(2, 1) and non-informative Gamma(0.001, 0.001) priors). These diagnostics are based on a representative simulation run with $n=60,$ $m=40,$ $p=0.3,$ and true parameters $(\alpha ,\lambda )=(1.0,0.8).$ Each figure contains three panels for each parameter or derived quantity:

- **Left panels (Trace plots):** These show the sampled values against iteration number. The chains exhibit rapid fluctuation around a stable mean without any visible trend or drift, indicating good mixing and convergence to the stationary distribution.
- **Middle panels (Posterior density estimates):** These display kernel density estimates of the posterior distributions. The densities are unimodal and approximately symmetric, supporting the asymptotic normality assumptions underlying the MLE-based CIs. The slightly narrower densities under the informative priors (Fig. 1) compared to the non-informative priors (Fig. 2) reflect the additional information incorporated.
- **Right panels (Running means):** These plot the cumulative mean against iteration number. The running means stabilize rapidly after the burn-in period (indicated by the vertical dashed line at 2, 000 iterations) and remain stable thereafter, confirming stationarity.

To supplement the visual diagnostics, we also compute quantitative convergence measures for all simulation replicates:

- **Effective sample size (ESS):** The ESS for $\alpha$ and $\lambda$ averages 5, 847 and 5, 621 across all scenarios, respectively, indicating that the 8, 000 post-burn-in samples provide information equivalent to at least 5, 600 independent draws.
- **Geweke diagnostic:**The Geweke z-scores (cf. Geweke^33^,) fall within the $(-2, 2)$ range for $97.3\%$ of replicates, indicating no evidence of non-convergence.
- **Heidelberger-Welch test:**The stationarity test (cf. Heidelberger and Welch^34^,) was passed by $98.1\%$ of chains at the $5\%$ significance level, further confirming convergence.

These diagnostics collectively demonstrate that the M–H algorithm performs reliably for the EpWD under progressive censoring.

#### Computational complexity

The computational efficiency of the proposed estimation methods was assessed using a standard desktop computer (Intel Core i7, 3.6 GHz, 16 GB RAM). For a representative simulation setting with $n = 120$, $m = 90$, and $p = 0.7$, each Monte Carlo replicate-incorporating both MLE and Bayesian estimation with 12, 000 MCMC iterations-required an average wall-clock time of approximately 3.64 seconds. The total execution time for a complete scenario of 1, 000 replicates is roughly 1 hour. Across various combinations of sample sizes and censoring schemes, the computational cost remained highly manageable, scaling approximately linearly with the sample size *n* and the number of iterations, demonstrating the feasibility of the developed algorithms for intensive reliability analysis.

#### Summary comparison of estimation methods

A systematic comparison of the three estimation methods across all simulation scenarios reveals consistent patterns:

- **MLE vs. Bayesian (overall):** Bayesian estimators uniformly outperform MLE in terms of lower MSE and better CP. The improvement is most pronounced for the shape parameter $\alpha$ and derived quantities (*R*, *h*, *J*, $J^{(\omega )}$), with MSE reductions of $8\%$–$15\%$ under moderate sample sizes ($n=60$) and heavy censoring ($p=0.7$, $m=40$).
- **Effect of sample size:** As sample size increases from $n=60$ to $n=120$, the performance gap narrows but Bayesian methods maintain an advantage of $3\%$–$7\%$ in MSE.
- **Effect of censoring:** Under light censoring ($p=0.3$), all methods perform similarly. Under heavy censoring ($p=0.7$), MLE exhibits noticeable under-coverage, while Bayesian credible intervals maintain near-nominal coverage.
- **Derived quantities:** The superiority of Bayesian methods extends to all derived quantities.

These findings establish the clear ordering $\text {B.Inf}> \text {B.Non-Inf}> \text {MLE}$ in terms of overall estimation performance, supporting the recommendation of Bayesian estimation with informative priors for practical applications of the EpWD under progressive censoring.

#### Impact of progressive type-II censoring

The simulation results across Tables 2–4 reveal consistent patterns regarding the impact of progressive type-II censoring:

- **Censoring severity:** Heavier censoring (smaller *m* relative to *n*, or $p=0.7$ versus $p=0.3$) uniformly increases MSE and reduces CPs for all methods. This effect is most pronounced for $\alpha$ and derived quantities.
- **MLE vulnerability:** MLE is most sensitive to censoring. Under heavy censoring ($p=0.7,$ $m=40$), MLE exhibits under-coverage (CP as low as $90.7\%$ for $\lambda$ in Table 2) and inflated MSE. For example, with $(\alpha ,\lambda )=(1.5,1.2)$ in Table 2, the MSE for $\alpha$ under MLE increases from 0.0313 ($n=120$) to 0.0630 ($n=60$) — a doubling of error.
- **Bayesian robustness:** Bayesian methods, particularly with informative priors, demonstrate greater resilience to censoring. Under the same heavy censoring scenario, Bayesian intervals maintain near-nominal coverage while achieving MSE reductions compared to MLE.
- **Bias patterns:** Censoring introduces systematic bias, particularly in MLE estimates of *h*(2) and extropy measures. In Table 3 with $n=60,$ $m=40,$ and $p=0.7,$ the MLE estimate for *h* shows $10.2\%$ relative bias versus $4.7\%$ for Bayesian informative priors.
- **Practical implications:** When heavy censoring is unavoidable, Bayesian methods with informative priors provide more reliable inference. The real data applications in Table 8 corroborate these patterns.

### Reproducibility and computational implementation

To ensure full reproducibility of all simulation results presented in this study, the complete source code has been prepared and documented. The code is written in the R programming environment (R Core Team^26^,) and includes the following components: data generation, MLEs, implementation of the Newton-Raphson algorithm, Bayesian estimation, performance metrics, and table and figure generation.

**Availability:** Due to space constraints, the complete code is not included in the main manuscript. However, it is available as supplementary material upon request from the corresponding authors. Interested readers may contact tahersobh46@gmail.com to obtain the code files. Upon publication, we will also consider making the code publicly available through a permanent repository (e.g., Zenodo or Figshare) to facilitate wider access and long-term preservation.

This commitment to reproducibility ensures that all findings in this study can be independently verified and extended by other researchers.

**Table 2:** Summary results for $\alpha$ and $\lambda$

$\alpha = 1$				MLE						Bayesian				Bayesian Non-Inf					
*n*	$\lambda$	*p*	*m*	Par	Est	MSE	MRE	AL	CP	Est	MSE	MRE	AL	CP	Est	MSE	MRE	AL	CP
60	0.8	0.3	40	$\alpha$	1.0401	0.0268	0.1254	0.5819	94.5	1.0218	0.0242	0.1202	0.5672	93.9	1.0178	0.0245	0.1211	0.5700	94.1
				$\lambda$	0.7931	0.0198	0.1415	0.5443	91.4	0.8643	0.0288	0.1655	0.6340	95.9	0.8451	0.0264	0.1589	0.6184	95.9
			50	$\alpha$	1.0361	0.0192	0.1071	0.4947	94.4	1.0273	0.0179	0.1039	0.4867	94.5	1.0190	0.0178	0.1036	0.4869	94.6
				$\lambda$	0.7957	0.0121	0.1083	0.4299	92.5	0.8388	0.0152	0.1205	0.4744	95.6	0.8248	0.0141	0.1159	0.4671	95.3
		0.7	40	$\alpha$	1.0519	0.0291	0.1291	0.5914	92.5	1.0329	0.0257	0.1217	0.5766	93.0	1.0296	0.0263	0.1228	0.5786	92.7
				$\lambda$	0.7869	0.0204	0.1423	0.5376	90.8	0.8573	0.0285	0.1619	0.6273	95.4	0.8383	0.0270	0.1573	0.6101	94.8
			50	$\alpha$	1.0322	0.0198	0.1081	0.4953	94.5	1.0232	0.0185	0.1054	0.4875	94.6	1.0152	0.0183	0.1050	0.4877	93.7
				$\lambda$	0.7998	0.0133	0.1148	0.4352	91.9	0.8439	0.0170	0.1266	0.4817	95.1	0.8292	0.0158	0.1222	0.4726	94.8
	1.2	0.3	40	$\alpha$	1.0474	0.0273	0.1258	0.5859	94.8	1.0353	0.0244	0.1196	0.5682	94.4	1.0249	0.0246	0.1207	0.5743	94.4
				$\lambda$	1.1849	0.0445	0.1391	0.8061	90.7	1.2666	0.0536	0.1498	0.8886	95.5	1.2620	0.0583	0.1550	0.9168	94.8
			50	$\alpha$	1.0341	0.0186	0.1046	0.4943	94.6	1.0279	0.0175	0.1017	0.4853	94.5	1.0173	0.0172	0.1016	0.4867	94.1
				$\lambda$	1.1920	0.0302	0.1144	0.6453	92.1	1.2450	0.0343	0.1194	0.6945	94.5	1.2355	0.0347	0.1202	0.7009	94.2
		0.7	40	$\alpha$	1.0430	0.0246	0.1213	0.5862	96.5	1.0312	0.0220	0.1156	0.5692	96.2	1.0204	0.0222	0.1166	0.5732	96.0
				$\lambda$	1.1918	0.0435	0.1401	0.8153	90.7	1.2746	0.0537	0.1533	0.9013	96.3	1.2702	0.0588	0.1589	0.9258	95.3
			50	$\alpha$	1.0307	0.0172	0.1001	0.4947	95.8	1.0244	0.0161	0.0971	0.4856	95.7	1.0140	0.0160	0.0970	0.4865	95.1
				$\lambda$	1.1914	0.0285	0.1103	0.6482	91.8	1.2447	0.0325	0.1167	0.6976	95.4	1.2348	0.0329	0.1171	0.7031	95.1
120	0.8	0.3	70	$\alpha$	1.0279	0.0149	0.0947	0.4439	93.6	1.0150	0.0137	0.0914	0.4360	94.3	1.0151	0.0141	0.0925	0.4368	93.0
				$\lambda$	0.7960	0.0137	0.1161	0.4486	92.2	0.8418	0.0177	0.1299	0.4935	94.0	0.8292	0.0165	0.1259	0.4824	94.0
			90	$\alpha$	1.0153	0.0094	0.0765	0.3718	94.7	1.0091	0.0090	0.0751	0.3671	94.6	1.0057	0.0091	0.0752	0.3674	94.8
				$\lambda$	0.8011	0.0076	0.0871	0.3438	95.0	0.8279	0.0091	0.0932	0.3653	96.2	0.8197	0.0086	0.0911	0.3604	96.1
		0.7	70	$\alpha$	1.0273	0.0129	0.0869	0.4445	96.3	1.0148	0.0119	0.0840	0.4361	95.9	1.0141	0.0121	0.0847	0.4370	95.8
				$\lambda$	0.7924	0.0123	0.1092	0.4457	93.0	0.8375	0.0155	0.1219	0.4893	95.6	0.8257	0.0146	0.1182	0.4791	95.3
			90	$\alpha$	1.0207	0.0095	0.0771	0.3744	95.2	1.0146	0.0091	0.0754	0.3701	94.7	1.0112	0.0090	0.0752	0.3696	94.8
				$\lambda$	0.7966	0.0075	0.0869	0.3404	94.0	0.8230	0.0086	0.0927	0.3614	96.3	0.8149	0.0082	0.0908	0.3559	95.7
	1.2	0.3	70	$\alpha$	1.0277	0.0141	0.0927	0.4438	94.2	1.0194	0.0131	0.0895	0.4344	94.3	1.0148	0.0133	0.0905	0.4369	93.4
				$\lambda$	1.1939	0.0304	0.1139	0.6720	91.8	1.2488	0.0356	0.1225	0.7165	94.6	1.2438	0.0369	0.1242	0.7236	94.5
			90	$\alpha$	1.0272	0.0099	0.0781	0.3755	95.1	1.0227	0.0095	0.0765	0.3701	94.9	1.0176	0.0093	0.0761	0.3709	94.7
				$\lambda$	1.1877	0.0174	0.0871	0.5033	92.3	1.2201	0.0186	0.0888	0.5246	94.2	1.2148	0.0187	0.0894	0.5263	93.9
		0.7	70	$\alpha$	1.0238	0.0143	0.0940	0.4430	95.2	1.0155	0.0133	0.0914	0.4340	94.3	1.0109	0.0136	0.0924	0.4362	94.7
				$\lambda$	1.1864	0.0306	0.1156	0.6718	91.7	1.2412	0.0348	0.1221	0.7166	94.3	1.2361	0.0362	0.1240	0.7230	94.0
			90	$\alpha$	1.0220	0.0101	0.0787	0.3749	94.4	1.0174	0.0097	0.0771	0.3701	94.4	1.0125	0.0096	0.0772	0.3705	93.8
				$\lambda$	1.1950	0.0172	0.0870	0.5101	92.4	1.2281	0.0189	0.0897	0.5336	94.8	1.2222	0.0189	0.0896	0.5344	94.8
α=1.5																			
60	0.8	0.3	40	α	1.5642	0.0533	0.1190	0.8751	96.0	1.5280	0.0453	0.1112	0.8464	95.6	1.5303	0.0481	0.1142	0.8566	95.5
				λ	0.7912	0.0086	0.0929	0.3576	92.2	0.8316	0.0108	0.1029	0.4047	95.9	0.8214	0.0101	0.0999	0.3958	95.3
			50	α	1.5518	0.0399	0.1036	0.7335	95.1	1.5299	0.0357	0.0990	0.7170	94.9	1.5265	0.0368	0.1005	0.7217	94.8
				λ	0.7921	0.0050	0.0716	0.2838	93.6	0.8160	0.0056	0.0751	0.3082	96.3	0.8088	0.0054	0.0735	0.3031	95.6
		0.7	40	α	1.5464	0.0544	0.1193	0.8691	94.6	1.5105	0.0476	0.1136	0.8410	94.5	1.5126	0.0501	0.1161	0.8511	93.9
				λ	0.7931	0.0092	0.0949	0.3648	91.4	0.8348	0.0119	0.1064	0.4140	95.4	0.8243	0.0111	0.1026	0.4045	95.3
			50	α	1.5435	0.0391	0.1021	0.7399	94.5	1.5222	0.0352	0.0976	0.7229	94.4	1.5183	0.0362	0.0992	0.7278	94.2
				λ	0.7955	0.0054	0.0737	0.2880	93.8	0.8198	0.0062	0.0780	0.3125	96.0	0.8127	0.0059	0.0763	0.3082	95.7
	1.2	0.3	40	α	1.5706	0.0616	0.1257	0.8786	94.5	1.5395	0.0525	0.1173	0.8476	94.3	1.5366	0.0555	0.1206	0.8594	94.3
				λ	1.1855	0.0227	0.0994	0.5360	90.3	1.2371	0.0262	0.1033	0.5909	94.1	1.2310	0.0268	0.1049	0.5935	93.8
			50	α	1.5378	0.0406	0.1036	0.7309	94.7	1.5189	0.0370	0.1000	0.7131	94.2	1.5126	0.0381	0.1015	0.7185	93.9
				λ	1.1919	0.0120	0.0735	0.4320	93.4	1.2235	0.0132	0.0765	0.4631	95.0	1.2175	0.0131	0.0764	0.4627	94.9
		0.7	40	α	1.5775	0.0630	0.1266	0.8864	94.2	1.5466	0.0535	0.1175	0.8555	94.8	1.5434	0.0567	0.1207	0.8675	94.0
				λ	1.1817	0.0195	0.0931	0.5335	92.2	1.2332	0.0224	0.0980	0.5891	95.0	1.2269	0.0229	0.0988	0.5907	94.6
			50	α	1.5487	0.0381	0.1022	0.7437	95.9	1.5292	0.0343	0.0974	0.7256	95.8	1.5231	0.0352	0.0988	0.7317	95.6
				λ	1.1892	0.0121	0.0743	0.4289	92.8	1.2207	0.0131	0.0769	0.4601	95.9	1.2151	0.0131	0.0766	0.4592	95.8
120	0.8	0.3	40	α	1.5428	0.0313	0.0915	0.6661	94.7	1.5197	0.0281	0.0875	0.6511	94.7	1.5238	0.0294	0.0894	0.6563	94.1
				λ	0.7949	0.0059	0.0758	0.2968	92.1	0.8213	0.0070	0.0826	0.3198	94.3	0.8145	0.0067	0.0805	0.3142	94.1
			50	α	1.5281	0.0219	0.0759	0.5593	95.2	1.5146	0.0205	0.0738	0.5498	94.9	1.5138	0.0209	0.0744	0.5531	94.8
				λ	0.7969	0.0035	0.0588	0.2268	92.6	0.8120	0.0038	0.0612	0.2377	94.9	0.8079	0.0037	0.0603	0.2355	94.3
		0.7	40	α	1.5421	0.0348	0.0957	0.6671	93.8	1.5188	0.0314	0.0917	0.6524	94.1	1.5224	0.0327	0.0936	0.6573	94.0
				λ	0.7901	0.0058	0.0768	0.2961	91.6	0.8165	0.0067	0.0810	0.3193	95.7	0.8099	0.0064	0.0797	0.3139	94.3
			50	α	1.5351	0.0236	0.0817	0.5633	94.4	1.5215	0.0220	0.0794	0.5545	94.6	1.5206	0.0224	0.0802	0.5572	94.1
				λ	0.7953	0.0037	0.0619	0.2257	92.4	0.8103	0.0040	0.0639	0.2370	95.2	0.8062	0.0039	0.0632	0.2342	94.4
	1.2	0.3	40	α	1.5429	0.0313	0.0916	0.6661	94.7	1.5242	0.0282	0.0876	0.6502	94.8	1.5238	0.0294	0.0894	0.6563	94.1
				λ	1.1924	0.0133	0.0758	0.4452	92.1	1.2264	0.0150	0.0806	0.4718	94.4	1.2217	0.0150	0.0805	0.4712	94.2
			50	α	1.5385	0.0239	0.0808	0.5632	95.1	1.5268	0.0223	0.0784	0.5538	95.1	1.5241	0.0227	0.0791	0.5563	95.4
				λ	1.1912	0.0076	0.0585	0.3369	93.3	1.2108	0.0080	0.0596	0.3496	95.2	1.2075	0.0080	0.0594	0.3491	95.1
		0.7	40	α	1.5319	0.0293	0.0904	0.6628	95.5	1.5135	0.0269	0.0872	0.6471	95.4	1.5125	0.0278	0.0886	0.6517	95.1
				λ	1.1936	0.0131	0.0757	0.4495	92.8	1.2279	0.0149	0.0790	0.4761	95.6	1.2238	0.0149	0.0793	0.4760	96.3
			50	α	1.5279	0.0212	0.0769	0.5609	96.1	1.5164	0.0199	0.0747	0.5518	95.9	1.5136	0.0203	0.0756	0.5544	95.9
				λ	1.1952	0.0071	0.0568	0.3406	94.4	1.2149	0.0076	0.0582	0.3540	96.1	1.2117	0.0076	0.0582	0.3536	96.2

**Table 3:** Summary results for *R*(*t*) and *h*(*t*)

$\alpha = 1$				MLE						Bayesian				Bayesian Non-Inf					
*n*	$\lambda$	*p*	*m*	Par	Est	MSE	MRE	AL	CP	Est	MSE	MRE	AL	CP	Est	MSE	MRE	AL	CP
60	0.8	0.3	40	*R*	0.1065	0.0024	0.3593	0.1802	89.9	0.1266	0.0026	0.3717	0.1904	93.8	0.1211	0.0024	0.3586	0.1853	93.9
				*h*	2.7403	1.3969	0.3219	3.6148	95.6	2.6445	1.2560	0.3082	3.3562	94.0	2.7003	1.3687	0.3192	3.4306	94.3
			50	*R*	0.1080	0.0015	0.2762	0.1485	90.9	0.1215	0.0016	0.2872	0.1526	94.3	0.1175	0.0015	0.2768	0.1492	94.0
				*h*	2.6036	0.6219	0.2303	2.5751	95.2	2.5373	0.5684	0.2218	2.4572	94.4	2.5583	0.5923	0.2258	2.4761	94.3
		0.7	40	*R*	0.1036	0.0024	0.3672	0.1780	88.7	0.1238	0.0025	0.3654	0.1894	93.1	0.1183	0.0024	0.3579	0.1835	92.8
				*h*	2.8092	1.4800	0.3409	3.7445	96.3	2.7096	1.3068	0.3220	3.4711	94.4	2.7722	1.4547	0.3376	3.5463	95.1
			50	*R*	0.1095	0.0016	0.2920	0.1493	90.4	0.1231	0.0018	0.3022	0.1537	93.3	0.1190	0.0017	0.2919	0.1500	93.4
				*h*	2.5846	0.6271	0.2348	2.5682	94.3	2.5179	0.5750	0.2274	2.4543	93.0	2.5402	0.5954	0.2306	2.4716	93.2
	1.2	0.3	40	*R*	0.2324	0.0039	0.2050	0.2304	92.3	0.2475	0.0035	0.1939	0.2259	94.2	0.2439	0.0036	0.1970	0.2275	93.7
				*h*	1.7170	0.3517	0.2613	1.8907	96.8	1.6729	0.3074	0.2461	1.7660	95.1	1.6799	0.3307	0.2552	1.8022	95.0
			50	*R*	0.2376	0.0027	0.1704	0.1916	92.5	0.2486	0.0026	0.1662	0.1900	92.9	0.2447	0.0026	0.1661	0.1900	93.1
				*h*	1.6355	0.1552	0.1898	1.3478	95.0	1.6007	0.1399	0.1817	1.2919	93.7	1.6042	0.1444	0.1849	1.3051	93.8
		0.7	40	*R*	0.2344	0.0038	0.2056	0.2313	92.9	0.2495	0.0034	0.1957	0.2271	95.5	0.2460	0.0036	0.1995	0.2280	94.7
				*h*	1.6968	0.3148	0.2566	1.8709	96.8	1.6544	0.2780	0.2432	1.7498	95.4	1.6603	0.2977	0.2520	1.7803	94.7
			50	*R*	0.2368	0.0025	0.1618	0.1919	93.5	0.2479	0.0023	0.1581	0.1900	95.0	0.2439	0.0023	0.1578	0.1899	95.0
				*h*	1.6326	0.1492	0.1870	1.3512	95.5	1.5978	0.1337	0.1795	1.2952	94.2	1.6020	0.1392	0.1828	1.3061	94.3
120	0.8	0.3	70	*R*	0.1079	0.0017	0.3013	0.1560	90.1	0.1214	0.0018	0.3077	0.1588	92.8	0.1176	0.0017	0.3011	0.1554	92.6
				*h*	2.6115	0.7007	0.2493	2.7785	95.9	2.5521	0.6451	0.2419	2.6423	94.6	2.5962	0.7029	0.2496	2.6848	94.4
			90	*R*	0.1106	0.0010	0.2225	0.1215	93.1	0.1190	0.0010	0.2282	0.1229	94.9	0.1165	0.0010	0.2233	0.1211	94.6
				*h*	2.4752	0.2691	0.1659	1.9507	94.4	2.4373	0.2559	0.1630	1.8928	93.3	2.4530	0.2623	0.1645	1.9025	93.8
		0.7	70	*R*	0.1070	0.0015	0.2823	0.1569	92.3	0.1204	0.0016	0.2868	0.1591	94.6	0.1168	0.0015	0.2805	0.1560	94.3
				*h*	2.5999	0.6068	0.2281	2.7667	96.5	2.5435	0.5626	0.2208	2.6308	95.8	2.5816	0.6018	0.2274	2.6671	95.9
			90	*R*	0.1088	0.0010	0.2255	0.1209	92.5	0.1172	0.0010	0.2288	0.1223	95.4	0.1148	0.0010	0.2250	0.1203	94.8
				*h*	2.5075	0.2867	0.1730	1.9857	96.3	2.4700	0.2700	0.1690	1.9270	95.6	2.4862	0.2796	0.1712	1.9350	95.4
	1.2	0.3	70	*R*	0.2361	0.0025	0.1628	0.1893	92.3	0.2459	0.0023	0.1585	0.1862	93.7	0.2436	0.0024	0.1597	0.1868	93.0
				*h*	1.6406	0.1809	0.2004	1.4701	95.5	1.6162	0.1664	0.1938	1.4066	94.3	1.6229	0.1759	0.1987	1.4250	93.9
			90	*R*	0.2372	0.0016	0.1291	0.1484	92.9	0.2438	0.0015	0.1260	0.1470	93.7	0.2417	0.0015	0.1266	0.1471	93.8
				*h*	1.6131	0.0929	0.1462	1.0673	95.2	1.5937	0.0868	0.1418	1.0365	94.5	1.5960	0.0888	0.1436	1.0439	94.5
		0.7	70	*R*	0.2333	0.0025	0.1644	0.1895	92.2	0.2431	0.0023	0.1576	0.1863	94.2	0.2408	0.0024	0.1596	0.1870	93.3
				*h*	1.6480	0.1825	0.2071	1.4843	95.7	1.6240	0.1686	0.2001	1.4209	94.9	1.6305	0.1777	0.2051	1.4389	94.9
			90	*R*	0.2392	0.0015	0.1277	0.1488	93.9	0.2459	0.0015	0.1255	0.1478	94.5	0.2436	0.0015	0.1255	0.1476	94.0
				*h*	1.5938	0.0831	0.1424	1.0548	96.2	1.5742	0.0776	0.1385	1.0270	94.9	1.5775	0.0798	0.1403	1.0323	94.9
α=1.5																			
	1.2	0.3	70	*R*	0.2661	0.0044	0.1909	0.2327	91.9	0.2803	0.0040	0.1802	0.2300	92.5	0.2766	0.0040	0.1826	0.2294	91.8
				*h*	2.3208	0.5938	0.2580	2.4501	94.7	2.2431	0.5044	0.2399	2.2908	93.2	2.2678	0.5516	0.2500	2.3338	93.1
			90	*R*	0.2716	0.0026	0.1461	0.1967	93.8	0.2812	0.0024	0.1427	0.1952	94.0	0.2779	0.0024	0.1426	0.1945	94.0
				*h*	2.1920	0.2518	0.1760	1.7213	96.6	2.1373	0.2244	0.1696	1.6512	95.4	2.1494	0.2342	0.1724	1.6660	95.6
		0.7	40	*R*	0.2648	0.0040	0.1814	0.2336	92.9	0.2791	0.0035	0.1711	0.2307	94.5	0.2753	0.0036	0.1725	0.2303	94.0
				*h*	2.3385	0.6280	0.2570	2.4817	96.2	2.2608	0.5298	0.2402	2.3207	95.3	2.2865	0.5886	0.2497	2.3680	94.8
			50	*R*	0.2712	0.0027	0.1512	0.1968	93.3	0.2807	0.0025	0.1472	0.1952	95.1	0.2776	0.0025	0.1471	0.1946	94.5
				*h*	2.2092	0.2398	0.1747	1.7516	96.3	2.1543	0.2129	0.1673	1.6807	95.0	2.1657	0.2216	0.1701	1.6952	95.1
120	0.8	0.3	70	*R*	0.0818	0.0013	0.3448	0.1370	89.2	0.0943	0.0014	0.3587	0.1418	93.5	0.0915	0.0014	0.3495	0.1388	93.0
				*h*	4.4421	2.1141	0.2536	4.9249	96.0	4.3434	1.9279	0.2455	4.6601	94.4	4.4244	2.1312	0.2545	4.7624	95.2
			90	*R*	0.0826	0.0008	0.2619	0.1073	90.6	0.0902	0.0008	0.2687	0.1087	93.8	0.0886	0.0008	0.2636	0.1073	93.1
				*h*	4.2475	0.9767	0.1780	3.5108	95.8	4.1819	0.9153	0.1738	3.3951	95.3	4.2134	0.9554	0.1764	3.4239	95.2
		0.7	70	*R*	0.0797	0.0013	0.3496	0.1352	89.2	0.0923	0.0014	0.3539	0.1405	93.4	0.0896	0.0013	0.3479	0.1377	92.4
				*h*	4.4896	2.2775	0.2646	5.0065	96.0	4.3893	2.0710	0.2543	4.7441	94.4	4.4674	2.2811	0.2649	4.8381	94.7
			90	*R*	0.0817	0.0008	0.2804	0.1067	90.1	0.0893	0.0009	0.2824	0.1083	94.1	0.0877	0.0008	0.2784	0.1067	93.0
				*h*	4.2953	1.0289	0.1921	3.5694	95.8	4.2297	0.9600	0.1873	3.4559	95.7	4.2609	1.0043	0.1903	3.4806	94.9
	1.2	0.3	70	*R*	0.2703	0.0026	0.1428	0.1893	92.1	0.2794	0.0024	0.1401	0.1868	93.3	0.2770	0.0024	0.1402	0.1869	92.9
				*h*	2.2216	0.3122	0.1921	1.9092	95.8	2.1793	0.2827	0.1853	1.8277	94.1	2.1965	0.3025	0.1902	1.8529	94.1
			90	*R*	0.2723	0.0016	0.1142	0.1507	94.1	0.2781	0.0015	0.1123	0.1495	93.9	0.2763	0.0015	0.1121	0.1493	94.5
				*h*	2.1747	0.1470	0.1414	1.3780	96.4	2.1438	0.1355	0.1372	1.3397	95.7	2.1514	0.1403	0.1388	1.3484	95.9
		0.7	70	*R*	0.2703	0.0023	0.1394	0.1899	94.3	0.2794	0.0022	0.1344	0.1874	95.4	0.2772	0.0022	0.1352	0.1875	94.7
				*h*	2.1980	0.2509	0.1836	1.8888	96.2	2.1569	0.2290	0.1770	1.8078	95.1	2.1712	0.2418	0.1812	1.8308	95.9
			90	*R*	0.2737	0.0014	0.1106	0.1509	95.6	0.2795	0.0014	0.1088	0.1498	95.2	0.2777	0.0014	0.1087	0.1496	95.7
				*h*	2.1488	0.1283	0.1316	1.3621	96.2	2.1188	0.1194	0.1283	1.3266	95.5	2.1259	0.1231	0.1301	1.3338	95.6

**Table 4:** Summary results for *J* and $J^{(\omega )}$

$\alpha = 1$				MLE						Bayesian				Bayesian Non-Inf					
*n*	$\lambda$	*p*	*m*	Par	Est	MSE	MRE	AL	CP	Est	MSE	MRE	AL	CP	Est	MSE	MRE	AL	CP
60	0.8	0.3	40	*J*	-0.5459	0.0120	0.1396	0.3469	95.3	-0.5975	2.9290	0.2635	0.4391	96.9	-0.5742	0.0558	0.1797	0.4691	96.3
				$J^{(\omega )}$	-0.1378	0.0005	0.1254	0.0771	94.5	-0.1354	0.0004	0.1202	0.0752	93.9	-0.1349	0.0004	0.1211	0.0755	94.1
			50	*J*	-0.5325	0.0058	0.1144	0.2935	95.3	-0.5385	0.0075	0.1225	0.3250	96.0	-0.5531	0.0095	0.1352	0.3448	96.3
				$J^{(\omega )}$	-0.1373	0.0003	0.1071	0.0656	94.4	-0.1361	0.0003	0.1039	0.0645	94.5	-0.1350	0.0003	0.1036	0.0645	94.6
		0.7	40	*J*	-0.5470	0.0094	0.1370	0.3400	93.7	-0.5374	0.2101	0.1771	0.4453	95.8	-0.5750	0.0338	0.1754	0.4913	95.4
				$J^{(\omega )}$	-0.1394	0.0005	0.1291	0.0784	92.5	-0.1369	0.0005	0.1217	0.0764	93.0	-0.1364	0.0005	0.1228	0.0767	92.7
			50	*J*	-0.5337	0.0072	0.1200	0.3002	93.2	-0.5407	0.0145	0.1326	0.3592	94.5	-0.5536	0.0128	0.1430	0.3845	94.7
				$J^{(\omega )}$	-0.1368	0.0003	0.1081	0.0656	94.5	-0.1356	0.0003	0.1054	0.0646	94.6	-0.1345	0.0003	0.1050	0.0646	93.7
	1.2	0.3	40	*J*	-0.3621	0.0037	0.1342	0.2194	94.1	-0.3667	0.0051	0.1469	0.2483	96.3	-0.3831	0.0254	0.1786	0.2697	95.7
				$J^{(\omega )}$	-0.1388	0.0005	0.1258	0.0776	94.8	-0.1372	0.0004	0.1196	0.0753	94.4	-0.1358	0.0004	0.1207	0.0761	94.4
			50	*J*	-0.3564	0.0030	0.1194	0.1968	94.1	-0.3623	0.0038	0.1282	0.2180	95.1	-0.3700	0.0049	0.1382	0.2319	95.8
				$J^{(\omega )}$	-0.1370	0.0003	0.1046	0.0655	94.6	-0.1362	0.0003	0.1017	0.0643	94.5	-0.1348	0.0003	0.1016	0.0645	94.1
		0.7	40	*J*	-0.3598	0.0035	0.1335	0.2185	95.4	-0.3674	0.0082	0.1519	0.2600	96.4	-0.3731	0.0065	0.1589	0.2853	96.3
				$J^{(\omega )}$	-0.1382	0.0004	0.1213	0.0777	96.5	-0.1366	0.0004	0.1156	0.0754	96.2	-0.1352	0.0004	0.1166	0.0760	96.0
			50	*J*	-0.3560	0.0027	0.1092	0.1969	95.6	-0.3544	0.0478	0.1335	0.2271	97.1	-0.3705	0.0080	0.1323	0.2492	97.0
				$J^{(\omega )}$	-0.1366	0.0003	0.1001	0.0655	95.8	-0.1357	0.0003	0.0971	0.0643	95.7	-0.1344	0.0003	0.0970	0.0645	95.1
120	0.8	0.3	70	*J*	-0.5316	0.0045	0.0989	0.2435	94.6	-0.5310	0.0045	0.0982	0.2556	95.3	-0.5411	0.0078	0.1064	0.2597	94.6
				$J^{(\omega )}$	-0.1362	0.0003	0.0947	0.0588	93.6	-0.1345	0.0002	0.0914	0.0578	94.3	-0.1345	0.0002	0.0925	0.0579	93.0
			90	*J*	-0.5242	0.0028	0.0812	0.2094	96.2	-0.5253	0.0029	0.0820	0.2196	96.4	-0.5322	0.0033	0.0861	0.2244	96.9
				$J^{(\omega )}$	-0.1345	0.0002	0.0765	0.0493	94.7	-0.1337	0.0002	0.0751	0.0486	94.6	-0.1333	0.0002	0.0752	0.0487	94.8
		0.7	70	*J*	-0.5313	0.0042	0.0968	0.2417	95.3	-0.5298	0.0041	0.0954	0.2524	95.8	-0.5379	0.0046	0.1008	0.2563	95.6
				$J^{(\omega )}$	-0.1361	0.0002	0.0869	0.0589	96.3	-0.1345	0.0002	0.0840	0.0578	95.9	-0.1344	0.0002	0.0847	0.0579	95.8
			90	*J*	-0.5252	0.0029	0.0818	0.2081	95.8	-0.5262	0.0030	0.0827	0.2181	96.6	-0.5329	0.0033	0.0865	0.2226	96.7
				$J^{(\omega )}$	-0.1352	0.0002	0.0771	0.0496	95.2	-0.1344	0.0002	0.0754	0.0490	94.7	-0.1340	0.0002	0.0752	0.0490	94.8
	1.2	0.3	70	*J*	-0.3539	0.0019	0.0972	0.1618	94.3	-0.3552	0.0019	0.0969	0.1684	95.3	-0.3590	0.0021	0.1012	0.1722	94.7
				$J^{(\omega )}$	-0.1362	0.0002	0.0927	0.0588	94.2	-0.1351	0.0002	0.0895	0.0576	94.3	-0.1345	0.0002	0.0905	0.0579	93.4
			90	*J*	-0.3510	0.0014	0.0838	0.1378	94.5	-0.3526	0.0014	0.0845	0.1435	95.4	-0.3558	0.0016	0.0880	0.1468	94.8
				$J^{(\omega )}$	-0.1361	0.0002	0.0781	0.0498	95.1	-0.1355	0.0002	0.0765	0.0490	94.9	-0.1348	0.0002	0.0761	0.0491	94.7
		0.7	70	*J*	-0.3571	0.0020	0.0993	0.1638	95.0	-0.3587	0.0020	0.0992	0.1708	95.7	-0.3625	0.0022	0.1043	0.1746	95.2
				$J^{(\omega )}$	-0.1357	0.0003	0.0940	0.0587	95.2	-0.1346	0.0002	0.0914	0.0575	94.3	-0.1339	0.0002	0.0924	0.0578	94.7
			90	*J*	-0.3503	0.0014	0.0839	0.1390	93.9	-0.3522	0.0014	0.0854	0.1456	95.2	-0.3556	0.0016	0.0891	0.1489	95.3
				$J^{(\omega )}$	-0.1354	0.0002	0.0787	0.0497	94.4	-0.1348	0.0002	0.0771	0.0490	94.4	-0.1342	0.0002	0.0772	0.0491	93.8
$$\alpha = 1.5$$																			
60	0.8	0.3	40	*J*	-0.4999	0.0050	0.1135	0.2582	96.3	-0.4879	0.0041	0.1049	0.2536	96.3	-0.4944	0.0044	0.1077	0.2547	96.7
				$J^{(\omega )}$	-0.2073	0.0009	0.1190	0.1160	96.0	-0.2025	0.0008	0.1112	0.1121	95.6	-0.2028	0.0008	0.1142	0.1135	95.5
			50	*J*	-0.4942	0.0028	0.0850	0.1966	96.1	-0.4876	0.0024	0.0804	0.1967	96.6	-0.4919	0.0026	0.0822	0.1969	96.5
				$J^{(\omega )}$	-0.2056	0.0007	0.1036	0.0972	95.1	-0.2027	0.0006	0.0990	0.0950	94.9	-0.2023	0.0006	0.1005	0.0956	94.8
		0.7	40	*J*	-0.4986	0.0054	0.1148	0.2587	94.9	-0.4868	0.0045	0.1064	0.2547	95.5	-0.4934	0.0048	0.1087	0.2558	95.3
				$J^{(\omega )}$	-0.2049	0.0010	0.1193	0.1152	94.6	-0.2001	0.0008	0.1136	0.1114	94.5	-0.2004	0.0009	0.1161	0.1128	93.9
			50	*J*	-0.4919	0.0028	0.0866	0.1976	96.2	-0.4855	0.0025	0.0824	0.1975	96.5	-0.4898	0.0026	0.0837	0.1981	96.4
				$J^{(\omega )}$	-0.2045	0.0007	0.1021	0.0980	94.5	-0.2017	0.0006	0.0976	0.0958	94.4	-0.2012	0.0006	0.0992	0.0964	94.2
	1.2	0.3	40	*J*	-0.3352	0.0026	0.1224	0.1733	93.7	-0.3292	0.0022	0.1119	0.1695	94.8	-0.3315	0.0023	0.1159	0.1712	95.0
				$J^{(\omega )}$	-0.2081	0.0011	0.1257	0.1164	94.5	-0.2040	0.0009	0.1173	0.1123	94.3	-0.2036	0.0010	0.1206	0.1139	94.3
			50	*J*	-0.3282	0.0013	0.0877	0.1313	94.8	-0.3251	0.0012	0.0837	0.1309	95.7	-0.3269	0.0012	0.0851	0.1317	95.6
				$J^{(\omega )}$	-0.2038	0.0007	0.1036	0.0968	94.7	-0.2013	0.0006	0.1000	0.0945	94.2	-0.2004	0.0007	0.1015	0.0952	93.9
		0.7	40	*J*	-0.3359	0.0026	0.1185	0.1744	94.6	-0.3299	0.0021	0.1087	0.1705	95.7	-0.3322	0.0023	0.1121	0.1721	94.8
				$J^{(\omega )}$	-0.2090	0.0011	0.1266	0.1174	94.2	-0.2049	0.0009	0.1175	0.1133	94.8	-0.2045	0.0010	0.1207	0.1149	94.0
			50	*J*	-0.3291	0.0013	0.0872	0.1322	95.5	-0.3260	0.0011	0.0831	0.1317	96.2	-0.3276	0.0012	0.0846	0.1324	96.0
				$J^{(\omega )}$	-0.2052	0.0007	0.1022	0.0985	95.9	-0.2026	0.0006	0.0974	0.0961	95.8	-0.2018	0.0006	0.0988	0.0969	95.6
120	0.8	0.3	70	*J*	-0.4921	0.0034	0.0916	0.2095	94.7	-0.4839	0.0030	0.0877	0.2058	94.2	-0.4882	0.0031	0.0891	0.2067	94.3
				$J^{(\omega )}$	-0.2044	0.0005	0.0915	0.0883	94.7	-0.2014	0.0005	0.0875	0.0863	94.7	-0.2019	0.0005	0.0894	0.0870	94.1
			90	*J*	-0.4868	0.0018	0.0674	0.1551	94.5	-0.4825	0.0016	0.0654	0.1542	95.1	-0.4849	0.0017	0.0659	0.1542	94.6
				$J^{(\omega )}$	-0.2025	0.0004	0.0759	0.0741	95.2	-0.2007	0.0004	0.0738	0.0729	94.9	-0.2006	0.0004	0.0744	0.0733	94.8
		0.7	70	*J*	-0.4953	0.0034	0.0937	0.2113	95.4	-0.4870	0.0030	0.0885	0.2078	96.1	-0.4912	0.0032	0.0905	0.2083	95.1
				$J^{(\omega )}$	-0.2043	0.0006	0.0957	0.0884	93.8	-0.2012	0.0006	0.0917	0.0864	94.1	-0.2017	0.0006	0.0936	0.0871	94.0
			90	*J*	-0.4886	0.0019	0.0719	0.1560	94.8	-0.4842	0.0017	0.0694	0.1552	95.4	-0.4866	0.0018	0.0702	0.1549	95.4
				$J^{(\omega )}$	-0.2034	0.0004	0.0817	0.0746	94.4	-0.2016	0.0004	0.0794	0.0735	94.6	-0.2015	0.0004	0.0802	0.0738	94.1
	1.2	0.3	70	*J*	-0.3281	0.0015	0.0916	0.1397	94.7	-0.3240	0.0013	0.0875	0.1368	94.3	-0.3255	0.0014	0.0891	0.1378	94.4
				$J^{(\omega )}$	-0.2044	0.0005	0.0916	0.0883	94.7	-0.2020	0.0005	0.0876	0.0861	94.8	-0.2019	0.0005	0.0894	0.0870	94.1
			90	*J*	-0.3261	0.0008	0.0697	0.1039	95.7	-0.3240	0.0007	0.0673	0.1029	95.8	-0.3248	0.0008	0.0679	0.1032	95.8
				$J^{(\omega )}$	-0.2039	0.0004	0.0808	0.0746	95.1	-0.2023	0.0004	0.0784	0.0734	95.1	-0.2019	0.0004	0.0791	0.0737	95.4
		0.7	70	*J*	-0.3271	0.0013	0.0892	0.1395	96.3	-0.3231	0.0012	0.0848	0.1366	96.6	-0.3244	0.0012	0.0863	0.1374	96.0
				$J^{(\omega )}$	-0.2030	0.0005	0.0904	0.0878	95.5	-0.2005	0.0005	0.0872	0.0857	95.4	-0.2004	0.0005	0.0886	0.0863	95.1
			90	*J*	-0.3244	0.0007	0.0657	0.1036	96.4	-0.3223	0.0007	0.0636	0.1027	96.3	-0.3231	0.0007	0.0644	0.1029	96.7
				$J^{(\omega )}$	-0.2025	0.0004	0.0769	0.0743	96.1	-0.2009	0.0003	0.0747	0.0731	95.9	-0.2006	0.0004	0.0756	0.0735	95.9

## Application of real data

### Dataset I (Electronic device lifetimes)

In this application, we will examine actual engineering data provided by Wang^35^. This dataset consists of 18 observations of electronic device failure times (in hours): 5,11,21,31,46,75,98,122,145, 165,196,224,245,293,321,330,350, and 420.

**Fig. 3:**
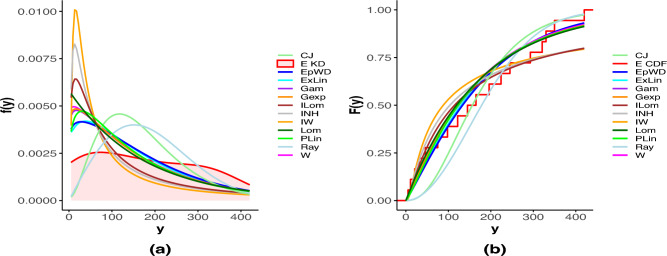
Fitted PDF and DF: EpWD vs theoretical distribution.

**Fig. 4:**
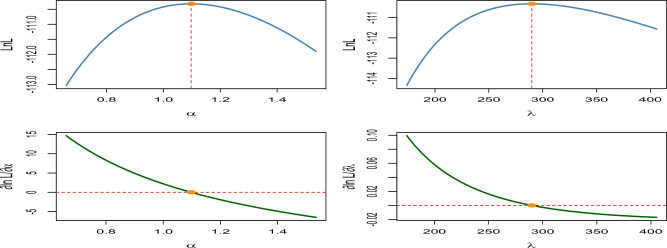
Log-profile likelihood plot for Dataset I.

**Fig. 5:**
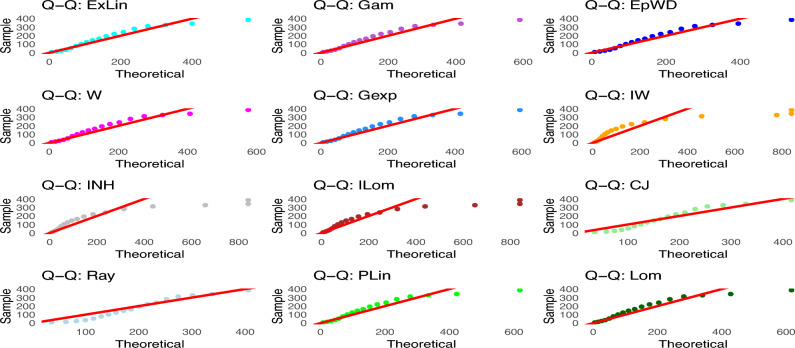
Q-Q plot comparisons of candidate distributions Dataset I.

Elshahhat et al.^36^ originally performed both maximum likelihood and Bayesian inference for the inverted Nadarajah–Haghighi model under a generalized type-II progressive hybrid censoring scheme, demonstrating accurate parameter estimation and tight interval estimates. In this paper the data are fitted using the EpWD with parameters $(\alpha , \lambda ).$ The MLEs of the parameters are $\widehat{\alpha } = 1.0976,$ and $\widehat{\lambda } = 289.8714.$ The EpWD model was compared with other models. Among all considered models, the EpWD yielded the lowest values for the AIC, corrected AIC (AICc), Bayesian information criterion (BIC), Hannan-Quinn information criterion (HQIC), and consistent AIC (CAIC), as well as the highest Kolmogorov-Smirnov (K-S) and *p*-value, confirming its superior goodness-of-fit. The results are presented in Table 5. Figures 3, 4, 5 further illustrate the EpWD’s superior alignment with empirical data through PDF, DF overlays, Q-Q plots, and log-profile likelihood visualizations. Collectively, these plots support the conclusion that the EpWD model provides a good fit to the data.

### Dataset II (Engineering data analysis)

This subsection examines the application of the suggested methodology to a real-world phenomenon through the analysis of a dataset provided by Lawless^37^. This dataset presents the cycle to failure statistics for 25 specimens of yarn, each measuring 100 cm, tested at a specific strain level: 15,20,38,42,61,76,86,98,121,146,149,157,175,176,180,180,198,220,224,251,264,282,321,325, and 653.

**Fig. 6:**
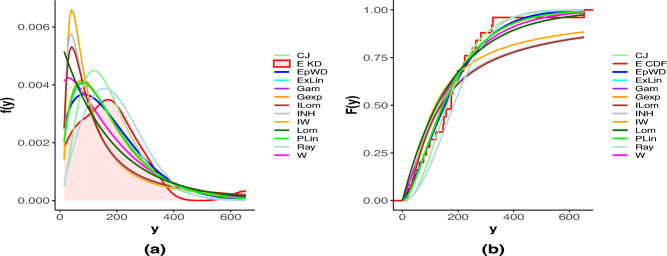
Fitted PDF and DF: EpWD vs theoretical distribution.

**Fig. 7:**
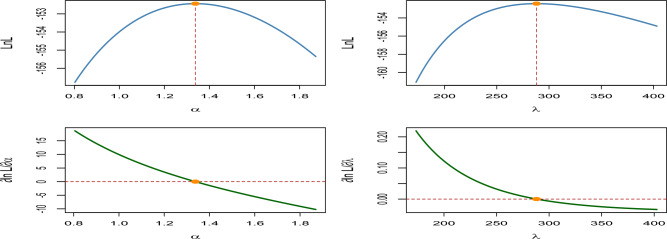
Log-profile likelihood plot for Dataset II.

**Fig. 8:**
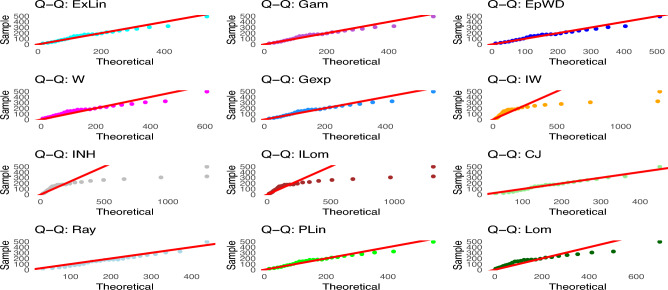
Q-Q plot comparisons of candidate distributions Dataset II.

Mohammed et al.^38,39^ originally used both frequentist and Bayesian MCMC methods to estimate the Xgamma distribution parameters and related reliability functions. When fitting the EpWD model, the MLE of the parameters are $\widehat{\alpha } = 1.3375$ and $\widehat{\lambda } = 288.0086.$ The EpWD model was compared with other models. Among all considered models, the EpWD yielded the lowest values for the AIC, AICc, BIC, HQIC, and CAIC, as well as the highest K-S and *p*-value, confirming its superior goodness-of-fit. The results are presented in Table 6. Figures 6, 7, 8 further illustrate the EpWD’s superior alignment with empirical data through PDF, DF overlays, Q-Q plots, and log-profile likelihood visualizations. Collectively, these plots support the conclusion that the EpWD model provides a good fit to the data.

The candidate distributions considered for comparison include: Exponential-Lindley (ExLin), gamma (Gam), generalized exponential (Gexp), power Lindley (PLin), Weibull (W), Lomax (Lom), inverse Nadarajah-Haghighi (INH), inverse Lomax (ILom), inverse Weibull (IW), Chris-Jerry (CJ), and Rayleigh (Ray).

**Comments on Tables** 7**and** 8: Table 7 presents progressively censored samples, with removal scheme $\mathcal R,$ from two real data sets under various censoring schemes. Each scheme is defined by the number of observed failures *m* and removal probability *p*, illustrating how censoring impacts the data structure and the amount of information available for inference. Table 8 reports estimation results for Dataset I using three methods: MLE, B. Inf, and B.Non-Inf. Estimates are provided for the model parameters $\alpha ,$ $\lambda ,$ reliability *R*(*t*), hazard function *h*(*t*), evaluated at $t=50$ (both *R*(50) and *h*(50) are abbreviated in Table 8 by *R* and *h*, respectively), and uncertainty measures (extropy *J* and WEX $J^{(\omega )}$). In Table 8, LB and UB denote the lower and upper bounds of the confidence interval. Bayesian methods, particularly with informative priors, yield narrower intervals and better coverage, highlighting their robustness under progressive censoring.

The estimated extropy and WEX values in Table 8 provide complementary insights into the fitted EpWD. For both datasets, *J* and $J^{(\omega )}$ are negative, with $|J^{(\omega )}| < |J|$ in most cases, reflecting the differential weighting of the upper tail. The narrower credible intervals for $J^{(\omega )}$ compared to *J* across all censoring schemes corroborate the simulation finding that WEX is more precisely estimable. This precision advantage makes $J^{(\omega )}$ a particularly useful summary for reliability applications where tail behavior is of interest.

The results in Table 8 further illustrate the robustness of the Bayesian approach to prior specification. For both datasets and across all censoring schemes, the estimates obtained under the informative Gamma(2, 1) priors and the non-informative Gamma(0.001, 0.001) priors are very similar. The differences in the point estimates for $\alpha$ and $\lambda$ are consistently less than $5\%$, and the credible intervals exhibit comparable coverage and length. This consistency between the two prior settings in real data applications mirrors the findings from the simulation study, reinforcing the conclusion that the posterior inferences are primarily driven by the data rather than by the prior assumptions.

#### Comparative performance in real data analysis

The relative performance patterns observed in the simulation study are corroborated by the real data results in Table 8. For both datasets and across all censoring schemes, Bayesian estimates (both informative and non-informative) yield narrower intervals than MLE while maintaining comparable or better coverage. The informative priors provide the most precise estimates, with credible intervals consistently shorter than those from non-informative priors, confirming the value of incorporating prior information even in practical applications.

**Table 5:** Comparative model fit analysis: EpWD vs competing distributions based on Dataset I.

**Models**	$\ln {L}$	**AIC**	**AICc**	**BIC**	**HQIC**	**CAIC**	**K-S Stat**	**p-value**
EpWD	−110.3180	224.6360	225.4360	226.4167	224.8815	228.4167	0.1087	0.9678
ExLin	−110.7590	225.5180	226.3180	227.2988	225.7636	229.2988	0.1155	0.9474
Gam	−110.6032	225.2064	226.0064	226.9872	225.4519	228.9872	0.1206	0.9283
Gexp	−110.6271	225.2542	226.0542	227.0349	225.4997	229.0349	0.1222	0.9216
PLin	−111.0309	226.0617	226.8617	227.8425	226.3073	229.8425	0.1247	0.9100
Lom	−110.6668	225.3337	226.1337	227.1144	225.5792	229.1144	0.1249	0.9091
W	−110.5271	225.0541	225.8541	226.8348	225.2996	228.8348	0.1268	0.9002
INH	−114.5524	233.1048	233.9048	234.8856	233.3504	236.8856	0.2004	0.4107
ILom	−114.2156	232.4313	233.2313	234.2120	232.6768	236.2120	0.2015	0.4040
IW	−116.3568	236.7136	237.5136	238.4943	236.9591	240.4943	0.2060	0.3775
CJ	−116.5833	235.1667	235.4167	236.0571	235.2895	237.0571	0.2288	0.2602
Ray	−115.3205	232.6409	232.8909	233.5313	232.7637	234.5313	0.2328	0.2429

**Table 6:** Comparative model fit analysis: EpWD vs competing distributions based on Dataset II.

**Models**	$\ln {L}$	**AIC**	**AICc**	**BIC**	**HQIC**	**CAIC**	**K-S Stat**	**p-value**
EpWD	−152.4368	308.8737	309.4192	311.3114	309.5498	313.3114	0.1193	0.8686
CJ	−154.4048	310.8095	310.9834	312.0284	311.1476	313.0284	0.1219	0.8513
ExLin	−152.4569	308.9138	309.4592	311.3515	309.5899	313.3515	0.1358	0.7457
PLin	−152.4657	308.9314	309.4769	311.3692	309.6075	313.3692	0.1380	0.7278
Gam	−152.4380	308.8760	309.4214	311.3137	309.5521	313.3137	0.1383	0.7255
Gexp	−152.4910	308.9820	309.5275	311.4198	309.6581	313.4198	0.1445	0.6739
Ray	−155.6584	313.3168	313.4907	314.5357	313.6549	315.5357	0.1561	0.5761
W	−153.3292	310.6585	311.2039	313.0962	311.3346	315.0962	0.1771	0.4131
Lom	−154.5942	313.1884	313.7338	315.6261	313.8645	317.6261	0.1986	0.2777
IW	−158.5789	321.1578	321.7033	323.5956	321.8340	325.5956	0.2111	0.2153
INH	−158.2082	320.4163	320.9618	322.8541	321.0925	324.8541	0.2178	0.1865
ILom	−157.9840	319.9679	320.5134	322.4057	320.6440	324.4057	0.2251	0.1588

**Table 7:** Progressively censored samples for Dataset I & II under various (*m*, *p*) schemes.

	**Scheme (***m* **, ** *p***)**		**Censored Times**
Dataset I	$(9, 0.3)$	$\underline{Y}$	5,31,75,98,165,224,321,330,350
		$\mathcal R$	5,3,0,1,0,0,0,0,0
	$(9, 0.7)$	$\underline{Y}$	5,11,75,122,145,224,293,330,350
		$\mathcal R$	4,2,3,0,0,0,0,0,0
	$(16, 0.3)$	$\underline{Y}$	5,21,31,46,98,122,145,165,196,224,245,293,321,330,350,420
		$\mathcal R$	2,0,0,0,0,0,0,0,0,0,0,0,0,0,0,0
	$(16, 0.7)$	$\underline{Y}$	5,11,21,31,46,75,98,122,145,165,196,293,321,330,350,420
		$\mathcal R$	0,0,2,0,0,0,0,0,0,0,0,0,0,0,0,0
Dataset II	$(14, 0.3)$	$\underline{Y}$	15, 20, 38, 42, 121, 146, 149, 157, 175, 198, 224, 321, 325, 653
		$\mathcal R$	5, 4, 0, 1, 0, 0, 1, 0, 0, 0, 0, 0, 0, 0
	$(14, 0.7)$	$\underline{Y}$	15, 20, 38, 42, 76, 121, 175, 180, 198, 251, 282, 321, 325, 653
		$\mathcal R$	6, 2, 3, 0, 0, 0, 0, 0, 0, 0, 0, 0, 0, 0
	$(22, 0.3)$	$\underline{Y}$	15, 38, 42, 61, 76, 98, 121, 146, 157, 175, 176, 180, 180, 198, 220, 224, 251, 264, 282, 321, 325, 653
		$\mathcal R$	2, 1, 0, 0, 0, 0, 0, 0, 0, 0, 0, 0, 0, 0, 0, 0, 0, 0, 0, 0, 0, 0
	$(22, 0.7)$	$\underline{Y}$	15, 20, 38, 42, 61, 76, 98, 121, 146, 157, 175, 176, 180, 180, 198, 220, 224, 251, 264, 321, 325, 653
		$\mathcal R$	1, 0, 2, 0, 0, 0, 0, 0, 0, 0, 0, 0, 0, 0, 0, 0, 0, 0, 0, 0, 0, 0

**Table 8:** Summary results for real data under progressive censoring.

	Dataset I	MLE			Bayesian			Bayesian Non-inf		
(*p*,*m*)	Par	Est	LB	UB	Est	LB	UB	Est	LB	UB
(0.3,9)	$\alpha$	1.2378	0.6162	1.8594	1.3471	0.7847	1.9688	1.1636	0.5866	1.7787
	$\lambda$	311.18	152.38	469.99	301.48	235.71	373.71	372.78	170.91	641.51
	*R*	0.8523	0.6929	1.0000	0.8541	0.7064	0.9703	0.8269	0.6661	0.9805
	*h*	0.0040	0.0008	0.0073	0.0039	0.0015	0.0065	0.0042	0.0011	0.0074
	*J*	−0.0012	−0.0018	−0.0006	−0.0014	−0.0017	−0.0010	−0.0013	−0.0022	−0.0005
	$J^{(\omega )}$	−0.1640	−0.2464	−0.0816	−0.1785	−0.2609	−0.1040	−0.1542	−0.2357	−0.0777
(0.7,9)	$\alpha$	1.1721	0.5767	1.7674	1.2928	0.7391	1.8860	1.0994	0.5315	1.6794
	$\lambda$	315.19	142.17	488.22	303.15	230.00	374.45	392.10	154.67	738.88
	*R*	0.8369	0.6726	1.0000	0.8429	0.7015	0.9722	0.8137	0.6440	0.9601
	*h*	0.0043	0.0010	0.0075	0.0041	0.0016	0.0066	0.0043	0.0014	0.0075
	*J*	−0.0012	−0.0019	−0.0006	−0.0014	−0.0018	−0.0010	−0.0017	−0.0024	−0.0004
	$J^{(\omega )}$	−0.1553	−0.2342	−0.0764	−0.1713	−0.2499	−0.0979	−0.1457	−0.2225	−0.0704
(0.3,16)	$\alpha$	1.2398	0.7179	1.7616	1.3072	0.8302	1.8007	1.1829	0.7025	1.6845
	$\lambda$	307.86	193.42	422.31	304.02	240.22	371.85	329.20	207.27	483.87
	*R*	0.8509	0.7119	0.9900	0.8511	0.7215	0.9595	0.8272	0.6788	0.9576
	*h*	0.0041	0.0013	0.0069	0.0040	0.0018	0.0063	0.0043	0.0018	0.0071
	*J*	−0.0012	−0.0017	−0.0008	−0.0013	−0.0016	−0.0010	−0.0013	−0.0019	−0.0007
	$J^{(\omega )}$	−0.1643	−0.2334	−0.0951	−0.1732	−0.2386	−0.1100	−0.1567	−0.2232	−0.0931
(0.7,16)	$\alpha$	1.0312	0.6181	1.4442	1.0847	0.6961	1.4851	0.9896	0.6039	1.4314
	$\lambda$	280.00	157.26	402.74	296.25	224.45	364.58	310.65	164.67	479.57
	*R*	0.7684	0.6008	0.9359	0.7861	0.6490	0.9166	0.7519	0.5693	0.9057
	*h*	0.0056	0.0025	0.0088	0.0051	0.0029	0.0072	0.0056	0.0026	0.0087
	*J*	−0.0014	−0.0021	−0.0008	−0.0015	−0.0020	−0.0010	−0.0016	−0.0030	−0.0007
	$J^{(\omega )}$	−0.1366	−0.1914	−0.0819	−0.1437	−0.1968	−0.0922	−0.1311	−0.1897	−0.0800
	**Dataset II**									
(0.3,14)	$\alpha$	1.1980	0.7580	1.6379	1.2568	0.8607	1.6918	1.1613	0.7454	1.6007
	$\lambda$	331.23	192.91	469.56	311.81	247.44	383.22	363.40	215.26	549.49
	*R*	0.8526	0.7290	0.9762	0.8460	0.7312	0.9508	0.8392	0.7081	0.9496
	*h*	0.0039	0.0014	0.0065	0.0041	0.0019	0.0061	0.0040	0.0016	0.0065
	*J*	−0.0012	−0.0016	−0.0007	−0.0013	−0.0016	−0.0010	−0.0012	−0.0017	−0.0006
	$J^{(\omega )}$	−0.1587	−0.2170	−0.1004	−0.1665	−0.2242	−0.1140	−0.1539	−0.2121	−0.0988
(0.7,14)	$\alpha$	1.1748	0.7403	1.6092	1.2249	0.8250	1.6415	1.1320	0.7099	1.5453
	$\lambda$	337.9481	194.6815	481.21	314.21	245.50	384.28	371.60	220.98	580.19
	*R*	0.8497	0.7230	0.9764	0.8392	0.7195	0.9441	0.8337	0.7063	0.9558
	*h*	0.0039	0.0013	0.0065	0.0042	0.0020	0.0062	0.0040	0.0016	0.0065
	*J*	−0.0011	−0.0016	−0.0007	−0.0013	−0.0016	−0.0010	−0.0012	−0.0018	−0.0007
	$J^{(\omega )}$	−0.1557	−0.2132	−0.0981	−0.1623	−0.2175	−0.1093	−0.1500	−0.2047	−0.0941
(0.3,22)	$\alpha$	1.4255	0.9797	1.8713	1.4516	1.0335	1.8619	1.3771	0.9690	1.8195
	$\lambda$	305.31	221.24	389.38	305.28	249.99	368.11	318.61	233.27	420.41
	*R*	0.8906	0.7994	0.9819	0.8869	0.7992	0.9608	0.8762	0.7749	0.9576
	*h*	0.0034	0.0012	0.0055	0.0034	0.0017	0.0053	0.0035	0.0016	0.0057
	*J*	−0.0012	−0.0016	−0.0009	−0.0013	−0.0015	−0.0010	−0.0012	−0.0016	−0.0009
	$J^{(\omega )}$	−0.1889	−0.2480	−0.1298	−0.1923	−0.2467	−0.1369	−0.1825	−0.2411	−0.1284
(0.7,22)	$\alpha$	1.3086	0.8972	1.7201	1.3304	0.9301	1.7512	1.2686	0.8760	1.6631
	$\lambda$	293.93	205.70	382.17	298.85	243.95	362.16	307.76	217.74	413.40
	*R*	0.8597	0.7540	0.9655	0.8579	0.7515	0.9442	0.8456	0.7299	0.9467
	*h*	0.0040	0.0017	0.0064	0.0040	0.0021	0.0060	0.0042	0.0020	0.0065
	*J*	−0.0013	−0.0017	−0.0009	−0.0013	−0.0016	−0.0010	−0.0013	−0.0017	−0.0009
	$J^{(\omega )}$	−0.1734	−0.2279	−0.1189	−0.1763	−0.2320	−0.1232	−0.1681	−0.2204	−0.1161

## Conclusion

This study presents Bayesian and non-Bayesian approaches for estimating the parameters of the EpWD derived from the $m-$GOS model. We assessed the efficacy of different estimators utilizing progressively type-II censored samples from the EpWD. The Bayesian estimates regularly surpassed the MLEs in terms of bias and MSE for all unknown parameters. We also computed the asymptotic CIs based on the MLE and offered explicit formulations for the FIM utilized in their development. Furthermore, we analyzed two prominent information metrics-extropy and WEX-for the EpWD. The practical efficacy of the EpWD was ultimately proved by its application to two real-world datasets. In all instances, the EpWD demonstrated a superior match relative to other established competing distributions.

Limitations of the EpWD model

- Inability to reduce to a single Weibull (which may be a disadvantage when a simpler model suffices).
- Common shape parameter $\alpha$ for both components, limiting flexibility.
- For extremely heavy tails, distributions like Pareto or inverse Weibull may be preferable.

## Data Availability

The datasets used and/or analysed during the current study are available from the corresponding author on reasonable request.

## Declarations

### Use of generative-AI tools declaration

The authors declare they have not used Artificial Intelligence (AI) tools in the creation of this article.
